# Soil plastispheres as hotspots of antibiotic resistance genes and potential pathogens

**DOI:** 10.1038/s41396-021-01103-9

**Published:** 2021-08-28

**Authors:** Dong Zhu, Jun Ma, Gang Li, Matthias C. Rillig, Yong-Guan Zhu

**Affiliations:** 1 State Key Laboratory of Urban and Regional Ecology, Research Center for Eco-Environmental Sciences, Chinese Academy of Sciences, Beijing, China; 2 Key Laboratory of Urban Environment and Health, Institute of Urban Environment, Chinese Academy of Sciences, Xiamen, China; 3 Freie Universität Berlin, Institute of Biology, Berlin, Germany; 4 Berlin-Brandenburg Institute of Advanced Biodiversity Research, Berlin, Germany

**Keywords:** Microbial ecology, Environmental sciences

## Abstract

In the Anthropocene, increasing pervasive plastic pollution is creating a new environmental compartment, the plastisphere. How the plastisphere affects microbial communities and antibiotic resistance genes (ARGs) is an issue of global concern. Although this has been studied in aquatic ecosystems, our understanding of plastisphere microbiota in soil ecosystems remains poor. Here, we investigated plastisphere microbiota and ARGs of four types of microplastics (MPs) from diverse soil environments, and revealed effects of manure, temperature, and moisture on them. Our results showed that the MPs select for microbial communities in the plastisphere, and that these plastisphere communities are involved in diverse metabolic pathways, indicating that they could drive diverse ecological processes in the soil ecosystem. The relationship within plastisphere bacterial zero-radius operational taxonomic units (zOTUs) was predominantly positive, and neutral processes appeared to dominate community assembly. However, deterministic processes were more important in explaining the variance in ARGs in plastispheres. A range of potential pathogens and ARGs were detected in the plastisphere, which were enriched compared to the soil but varied across MPs and soil types. We further found that the addition of manure and elevation of soil temperature and moisture all enhance ARGs in plastispheres, and potential pathogens increase with soil moisture. These results suggested that plastispheres are habitats in which an increased potential pathogen abundance is spatially co-located with an increased abundance of ARGs under global change. Our findings provided new insights into the community ecology of the microbiome and antibiotic resistome of the soil plastisphere.

## Introduction

Due to residues of agricultural use of plastic film [1, 2], use of organic fertilizers [3], irrigation of reclaimed water [4], atmospheric deposition [5, 6] and other pathways via which plastic arrives in ecosystems [7], a large amount of plastic debris is accumulating in soils and terrestrial ecosystems. The increase of plastic debris is becoming recognized as an emerging global environmental problem [8, 9]. Microplastics (MPs; <5 mm in size) are the main components of this plastic debris, and the content of MPs could be up to 6.7% in some surface soils [10]. An increasing number of studies has shown that the accumulation of MPs in the soil ecosystem could cause adverse effects on soil biota and alter soil properties and biogeochemical cycles [8, 9, 11–14]. For instance, Boots et al. [11] studied effects of different types of MPs on soil biophysical parameters. Their results indicated that MPs could affect plant health (e.g., reduction of biomass) and soil structure (e.g., alteration of soil aggregate stability). Recently the interaction between MPs and microbes has moved into focus [15–18]. The MP particles will provide a new niche for microbiota, when they enter in the environment [18]. Microbiota will colonize the surface of MPs, forming a biofilm, also termed as the plastisphere [15, 18]. Biofilm formation on MPs has recently been documented in aquatic ecosystems [15, 18], indicating that plastisphere could affect aquatic ecosystem functioning (e.g., carbon cycling, xenobiotic compound degradation [19] and gene exchange [20]). Although many studies have revealed the composition and diversity of plastisphere microbiota in aquatic ecosystems [17–19], our knowledge about plastisphere microbiota is still poor, especially for their function in the soil ecosystem. A recent study has revealed that the addition of MPs could enhance microbial turnover and nutrient efficiency in the plastisphere [21], however, it did not examine the microbial composition of the plastisphere, which restricts our understanding about the roles of plastisphere microbiota in the soil ecological function. Furthermore, microbial community assembly in the plastisphere from the soil community at large is not well understood so far. A review has suggested that theories of community ecology could contribute to explaining microbial community assembly in the plastisphere [18]. To fully understand how MPs are influencing soil ecosystems, more knowledge about microbial community assembly in the plastisphere is needed.

The spread of antibiotic resistance genes (ARGs) has become a global health crisis [22–24]. Evidence that MPs act as a reservoir and refuge for ARGs and potential pathogens, suggests that MPs have an important contribution to the spread of ARGs and could potentially affect disease transmission and treatment [25, 26]. Previous findings from laboratory experiments have indicated that the frequency of plasmid transfer between plastic-associated bacteria was higher than that in free-living bacteria [20]. However, these studies mainly focused on aquatic ecosystems [20, 25, 27, 28]. To date, only few studies have determined ARGs in the plastic surface in the soil ecosystem [29, 30]. These studies only focused on several types of ARGs and MPs, and the comprehensive study is also lacking. The antibiotic resistome of plastispheres in the soil ecosystem is thus still largely unknown, especially for diverse soil environments and different MP types, although soil has been recognized as an important reservoir of ARGs [23]. This currently limits our understanding of the role of soil MPs in the environmental dispersal of ARGs.

The bacterial community has a key role in determining the abundance and diversity of ARGs in multiple environments [31–33]. Assembly of the microbial community is commonly determined by deterministic and stochastic processes, currently a frontier of microbial ecology research [34]. Nonetheless, the contribution of the microbial community, as structured by deterministic and stochastic processes, to the variation of ARGs has rarely been studied. Previous studies have revealed a higher abundance of ARGs existing in the plastisphere compared to the surrounding environment [28, 30], indicating environmental selection (i.e., deterministic processes), and given the role of MPs as vectors for bacteria [35]. Therefore, we hypothesized that the contribution of deterministic processes acting on bacterial communities to the antibiotic resistome of the plastisphere is higher than that of stochastic process. Certainly, bacteria randomly impinging on the plastisphere may also carry ARGs, especially via horizontal transfer.

Soil is not only a “hot spot” for MPs, but is also suffering from various disturbances in the context of global change [36]. Among the most concerning factors are shifts in soil temperature and moisture caused by global change. Numerous studies have shown that temperature and moisture have an important influence on soil microbial community [37, 38], and in the study of compost, effects of temperature and moisture on ARGs have also been revealed [39–41]. Researchers further found that temperature and humidity affected microbiome and antibiotic resistome of building surfaces [42]. Since MPs are generally hydrophobic, the responses of plastisphere microbiome and resistome to temperature and moisture may be more sensitive compared to responses in the soil at large. In addition, manure is generally believed to be an important cause of the increase of ARGs in soil ecosystems [43]. Previous studies have indicated that application of manure could increase the abundance of ARGs in many interfaces (e.g., rhizosphere and phyllosphere) [44, 45]. Therefore, more studies are needed to decipher effects of manure, temperature and moisture on the plastisphere.

Thus, the objectives of our study were (1) to characterize the composition and diversity of microbial communities in different MP plastispheres from diverse soil environments, (2) to elucidate the interactions, community assembly mechanisms and potential functions of plastisphere microbiota, (3) to reveal the composition of potential bacterial pathogens and the antibiotic resistome in the plastisphere, (4) to explore the relationship between bacterial communities and antibiotic resistance genes and (5) to decipher effects of manure, temperature and moisture on the microbiome and antibiotic resistome of plastisphere, by employing amplicon and metagenomic sequencings and high-throughput qPCR techniques. Our findings will provide insights into the microbial community assembly and the ecological mechanisms underpinning a potential shift in ARGs in the plastisphere from the bulk soil.

## Materials and methods

### Soils and microplastics

Three different types of representative soils (Red soil, Yellow brown soil, and Black soil) were used in this study. The Red soil and Black soil were collected from arable lands of Yunnan (KM) and Heilongjiang (HA) provinces, China, respectively. At the same time, we collected Yellow brown soil from arable and forest lands of Jiangsu (NJ) province, China, respectively. After the soil samples were collected, we divided them into two parts. One part was used to analyze basic soil properties, and we used the other part to conduct the incubation of MPs after soil samples had been cleared of coarse materials (e.g., plant residues and rocks) by sieving (2 mm). Soil properties are showed in Table S1.

We used four different types of MPs in this study, purchased from Youngling Electromechanical Technology Co. (Shanghai, China). The four MP beads are polyvinyl chloride (PVC), polyamide (PA), polyethylene (PE) and polystyrene (PS) with the same particle size each (around 30 μm); these were chosen since they are among the most common plastic debris in soils [46, 47]. The specific surface areas, average adsorption pore widths and hydrophobicity (contact angle) of the four MPs are listed in Tables S2 and S3. Before the incubation of MPs, we used 1% sodium hypochlorite to treat these MPs for 30 min and sterile water to wash five times for disinfection. In addition, around 30 μm glass beads and different sizes of PE MPs (around 30, 200, and 1500 μm) were also included in this study.

### Experimental design

**Fig. 1:**
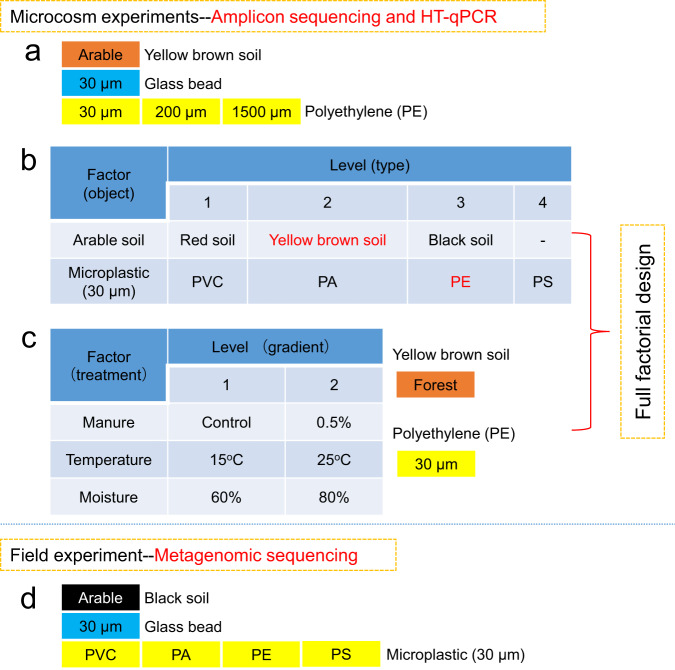
Experimental design. Three microcosm experiments (**a**, **b**, and **c**) and one field experiment (**d**) were performed in this study. In the first experiment (**a**), bacterial communities from 30 μm glass bead as a negative control and different size of PE (30, 200, and 1500 μm) were analyzed to exclude the mesh bag itself caused the selection for bacteria in the plastisphere. In the second experiment (**b**), we studied the responses of bacterial communities to different microplastics (PVC, PA, PE, and PS) plastisphere from diverse soil environments, identified the bacterial community assembly process in plastisphere, and examined the compositions of pathogens and antibiotic resistance genes as well as their relationships with the bacterial communities in plastisphere. In the third experiment (**c**), effects of manure, temperature, and moisture on the microbiome and antibiotic resistome in the PE plastisphere were studied. In the fourth experiment (**d**), the composition, function, and resistome of microbial communities in different plastispheres and glass bead were revealed by metagenomic analysis.

To systematically decipher the microbial composition, function, and antibiotic resistome of plastispheres, three microcosm experiments and one field experiment were performed in this study (Fig. 1). Since microplastics (30 μm) are incubated directly in the soil, they are difficult to collect. We used a method of nylon mesh bags (mesh size: 50 μm; similar to rhizosphere bags [48]) to study the plastisphere microbiota. Each nylon mesh bag only contained 1 g MPs, which was buried into the soil and then incubated for 8 weeks (see Fig. S1 for a schematic diagram of microplastics incubation). For the microcosm experiment, the MPs were incubated in the glass container (Length: 0.22 m, Width: 0.155 m, and Height: 0.108 m). Each glass container contained 2 kg dried soils, and each treatment was replicated five times. Before the incubation of MPs, we adjusted soil moisture content to 75% of field capacity, and these soils were incubated for two weeks to activate microbiota. In the process of the incubation of MPs, sterile water was added twice a week to keep soil moisture. At the end of the incubation period, soil and MP samples were collected and stored at −80 °C for further analysis. High-throughput qPCR was used to detect ARGs, and the bacterial community of plastispheres was analyzed by amplicon sequencing. For MPs, we collected nylon mesh bags from the pot and field, and then the nylon mesh bags were cut open to obtain MPs samples.

### Microcosm experiment 1 (Fig. 1a): validity of the method of nylon mesh bags

The 30 μm glass beads as a negative control and different size of PE MPs (30, 200, and 1500 μm) were transferred into nylon mesh bags, respectively, and then were incubated in the Yellow brown soil which was collected from arable land. We used glass beads in this experiment to exclude that just any 30 µm sized particle, irrespective of whether it is microplastic or not, would lead to similar effects on the microbial community due to a mesh bag effect.

### Microcosm experiment 2 (Fig. 1b): responses of bacterial communities and antibiotic resistome to different MPs plastispheres from diverse soil environments

The three different types of soils were transferred into glass containers. A total of 15 glass containers were set up to incubate MPs. Each type of MP was divided into 15 parts, which was then transferred into 15 sterile nylon mesh bags, and a total of 60 nylon mesh bags were used. Each glass container contained four nylon mesh bags, and these nylon mesh bags contained the different types of MPs. The MP samples were nested within experimental unit (the glass container).

### Microcosm experiment 3 (Fig. 1c): effects of manure, temperature and moisture on the microbiome and antibiotic resistome of plastisphere

We selected the full factorial design to study effects of three factors (manure, temperature, and moisture) on the microbiome and antibiotic resistome of 30 μm PE plastispheres. Each factor included two levels for a total of eight treatments (Fig. 1c). The PE MPs samples were incubated in the Yellow brown soil, which was collected from forest land. Since this experiment included the treatment with added manure, we used forest soil without added manure. The manure was obtained from a Bio-fertilizer co., LTD (Zhejiang, China). In the treatment with added manure, 5 g manure was mixed with per kg dry soil. The basic properties of manure are: total *N* = 30.2 mg kg^−1^, total C = 231 mg kg^−1^, ofloxacin = 0.028 mg kg^−1^, and oxytetracycline = 0.032 mg kg^−1^.

### Field experiment (Fig. 1d): metagenomic insights into the composition, function, and antibiotic resistome of microbial communities in different plastispheres

The 30 μm glass beads and PVC, PA, PE, and PS MPs were transferred into nylon mesh bags, respectively, and then were buried into the Black soil in the field. The site where the nylon mesh bag were buried was the same site from which the Black soil for the microcosm experiment was collected. Each treatment was replicated three times. After incubation for 8 weeks, soil, glass bead, and MPs samples were collected, and metagenomic sequencing was used to analyze their microbial composition, function, and the resistome.

### DNA Isolation

We used the FastDNA Spin Kit for soil (MP Biomedicals, France) to extract DNA from 0.5 g soils, glass beads and MPs, following the manufacturer’s instruction. Then we used spectrophotometric analysis (Nanodrop ND-1000, America) and 1.0% agarose gel electrophoresis to check the quality and concentration of isolated DNA. Finally, we stored the isolated DNA at −80 °C for further analysis.

### High-throughput qPCR for detection of antibiotic resistance genes

A total of 285 ARGs, 10 mobile genetic elements (MGEs, including eight transposases and two integrons) and the 16 S rRNA gene were determined in each soil and MP sample by using the SmartChip Real-Time PCR System (WaferGen Biosystems, Inc., America). Primers and PCR conditions for all gene amplifications were as given in previous studies [49, 50]. Each amplification of genes was carried out in three technical replicates. We used the SmartChip qPCR software to process the raw output data. Only when the three technical replicates had all been amplified and when the cycle number of amplification was lower than the threshold cycle (31), we took the gene as detected. The relative abundance of ARGs was used in this study, which was calculated according to the equation reported previously [49, 50].

### Amplicon sequencing and bioinformatic analysis

We used bacterial universal primer 515 F (5ʹ-GTGCCAGCMGCCGCGG-3ʹ) −806R (5ʹ-GGACTACHVGGGTWTCTAAT-3ʹ) to amplify the V4 region of 16 S rRNA gene [51]. The unique barcodes combined with primers were used to differentiate samples. The conditions of PCR and purification of products were consistent with previous studies [52, 53]. The concentration of purified products was determined using the Qubit 3.0 fluorimeter (Invitrogen). In our study, the 24 samples labeled with different barcodes of equal DNA content were pooled to a library for sequencing. For example, a total of four libraries were built in Experiment 2. The amplicon sequencing was performed in the MiSeq 300 instrument (Meiji biological medicine co., LTD, China).

After the quality of sequences was initially checked using FastQC, we used USEARCH v11.0.667 to analyze the obtained raw sequencing data, following the online instructions [54]. In brief, paired-end sequences were merged, primer sequences were removed, and we discarded sequences that have a predicted error rate of 1.0 bases per sequence. Afterwards, unique sequences were identified and denoised. The unoise3 algorithm was employed to pick zero-radius operational taxonomic units (zOTUs), and chimeras were filtered. Subsequently, we use the otutab command to make a zOTU table. The sequences of zOTUs were classified using the feature-classifier classify-sklearn command with the SILVA (v138) reference database in the QIIME2-2020.6 [55]. Finally, we removed zOTUs with <8 reads across all samples (including soils and MPs) before downstream analysis. The alpha (Shannon index) and beta diversity (Bray Curtis and Weighted UniFrac distances) of bacterial communities were calculated using QIIME2-2020.6. We identified potential bacterial pathogens by using the high-quality sequences to blast a reference database with the strict criteria of sequence identity >99% and E-value <1 × 10^−10^ which was reported in our previous studies [56, 57].

### Metagenomic sequencing and bioinformatic analysis

We used the Covaris M220 (Gene Company Limited, China) to fragment the obtained DNA, and then screened fragments of about 400 bp to construct the paired-end library via the NEXTFLEX Rapid DNA-Seq according to the manufacturer’s instructions. A HiSeq (Illumina Inc., San Diego, CA, USA) was selected to perform the metagenomic sequencing of paired-end library at the Majorbio Bio-Pharm Technology Co., Ltd. (Shanghai, China). For the obtained metagenomic data, we used the fastp [58] to perform quality clipping of adapter sequences from reads and remove low-quality of reads. The MEGAHIT was employed to assemble majorizing sequences, and then we selected the contigs (length > = 300 bp) for further gene prediction and functional annotation. We used MetaGene [59] to perform Open reading frames (ORFs) prediction of selected contigs. Genes (length > = 100 bp) were selected and translated into amino acid sequences. The CD-HIT was used to construct a non-redundant gene catalog, and the gene abundance was calculated by comparing high quality reads per sample with the constructed non-redundant gene catalog using SOAPaligner. We adopted RPKM (Reads Per Kilobase Million) to indicating the abundance of gene in this study. We obtained taxonomic, KEGG, carbohydrate-active enzymes and antibiotic resistance annotations by aligning representative sequences to NCBI NR, Kyoto Encyclopedia of Genes and Genomes, CAZy, and ARDB databases (e-value: 1e–5) using Diamond, respectively. Finally, two samples belonging to PVC MPs were discarded due to insufficient data, and a total of 16 metagenomic sequencing of data were used in our study.

### Neutral community model

In this study, we constructed a neutral community model to determine the assembly mechanism for the microbial community in the plastisphere. The neutral community model we used was built by Sloan et al. [60], and it made three assumptions for the assembly of the microbial community: (1) the zOTUs in the plastisphere were sampled randomly from a common pool; (2) death and growth rates of zOTUs were equivalent in the plastisphere (ecological fitness); and (3) dispersal rates of zOTUs were equivalent between plastispheres (immigration). Thus, this model assumed that stochastic changes of zOTU abundances caused variations of microbial communities in the plastisphere (ecological drift). In this model, the mean relative abundance of each zOTU across plastispheres was used to predict the occurrence frequency of that zOTU. We compared the difference of fitting effect between the present model and a binomial distribution model [61] based on Akaike’s information criterion. The binomial distribution model is a simpler neutral model, assuming that zOTUs present a binomial distribution in the plastisphere. We used the R code of Burns et al. [62] to calculate the contribution of stochastic process to the microbial assembly of the plastisphere based on the rarefied zOTUs matrix.

### Statistical analysis

The composition of microbial community and relative abundance of ARGs from high-throughput qPCR were presented as mean values. The Shannon index of microbial community, ratio of potential bacterial pathogens/bacteria detected, relative abundance of MGEs and abundance of ARGs from metagenomics sequencing were presented as mean value ± standard error (SE). We employed the package vegan 2.5-6 to perform PERMANOVA using the Adonis test (999 permutations) in R version 4.0.3 [63], which revealed the difference of microbial community and ARGs between different treatments and the contribution of soil and MPs types, manure, temperature and moisture to variations of microbial community and ARGs in the plastisphere. The psych package of R was used to calculate the Spearman correlations between prevalent bacterial zOTUs (present in at least 80% samples) and between bacterial phylum and ARGs. The significant relationships between prevalent bacterial zOTUs and between bacterial phylum and ARGs were visualized in co-occurrence networks using the Gephi 0.9.2. The contribution of deterministic and stochastic processes acting on bacterial communities to changes of ARGs in the plastisphere was also assessed through Procrustes test and partial redundancy analysis, which were performed in the vegan 2.5–6 and labdsv 2.0–1 packages of R. Metabolic pathways of bacterial communities in different plastispheres were predicted by Tax4Fun2 [64]. The bubble diagram was made by the gglopt2 package of R. Venn diagram was selected to present shared microbial zOTUs and ARGs between different treatments. Significant level was set at *p* < 0.05. We used the Origin 2017 to produce other graphics in this study.

## Results

### Validity of the study based on the microcosm experiment 1

**Fig. 2:**
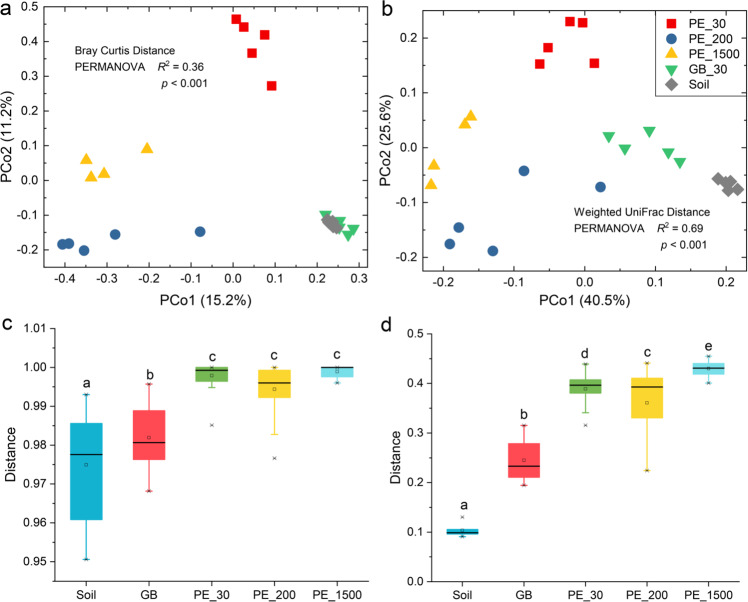
Structure of bacterial community. Principal coordinates analysis (PCoA) presented the distribution of bacterial communities from different substrates (soil, glass bead (GB: 30 μm) and polyethylene microplastics (PE: 30, 200, and 1500 μm)) based on the Bray Curtis (**a**) and Weighted Unifrac (**b**) distances. Different shapes and colors represented different types of samples. The variation explained by the PCoA axes was listed in parentheses. The PERMANOVA was used to test significant difference (significant level *p* < 0.05). Boxplots revealed the distance of bacterial communities between each substrate and soil sample (**c**: Bray Curtis and **d**: Weighted Unifrac), which reflected the similarity of bacterial community between each substrate and soil sample. Significance of results was evaluated using pairwise PERMANOVA and labeled using different letters. center line, median; box limits, first and third quartiles; whiskers, 1.5× interquartile range.

In the microcosm experiment 1, we obtained 341,304 sequences and 4650 bacterial zOTUs at 100% identity. The bacterial zOTUs table was rarefied to 6721 reads per sample. Bacterial composition of PE plastisphere was obviously different from glass bead and soil at the phylum, family, and genus levels (Figs. S2 and S3), and alpha diversity of PE plastisphere was overall lower than glass bead and soil (Fig. S4). Bacterial composition and alpha diversity of different size of PE plastisphere were also different. Principal coordinates analysis (PCoA) showed that plastisphere samples were clustered based on the sample size, and obviously separated from glass bead and soil samples (PERMANOVA, *p* < 0.001; Fig. 2a and b). We further found that structures of bacterial community between each substrate and soil sample were remarkably different, and the similarity between glass bead and soil sample was significantly higher than that between plastisphere and soil (pairwise PERMANOVA, *p* < 0.05; Fig. 2c and d). These results provided evidence that the microplastic we added is a factor that selects bacterial community in the plastisphere, and the selectivity is related to the properties of microplastics (e.g., the size of microplastics).

### Composition and diversity of bacterial community in the plastisphere based on the microcosm experiment 2

In microcosm experiment 2, we obtained 3,286,303 sequences and 5774 bacterial zOTUs at 100% identity. The bacterial zOTUs table was rarefied to 17701 reads per sample. The phyla Proteobacteria (51.3%) and Actinobacteria (30.1%) dominated the bacterial community of the plastisphere (Fig. S5a). Compared with other MPs, a higher abundance of the phylum Firmicutes was detected in the plastisphere of PS MPs (15.6%). The composition of bacterial communities in the plastisphere was different from that in the soil environment (Fig. S5a). For example, the relative abundance of the dominant phylum Proteobacteria in the plastisphere was significantly higher than that in the soil environment (*p* < 0.001), by 115%. Higher relative abundances of the phyla Acidobacteria and Chloroflexi were observed in the soil environment compared to the plastisphere (*p* < 0.001).

The bacterial Shannon index of plastisphere was markedly lower than that of the soil (*p* < 0.001; Fig. S5b), by 31.4%. The Shannon index of bacteria was not significantly different between different plastispheres from diverse soil environments (*p* > 0.05; Fig. S5b). Principal coordinates analysis of the bacterial community revealed that plastisphere samples were clearly separated from soil samples along PCo1 axis, and some clustering of plastisphere samples by MPs and soil types was also observed (PERMANOVA, *p* < 0.001; Fig. S6). Overall, PERMANOVA tests further confirmed that the MPs and soil types all significantly contributed to the differentiation of plastisphere bacterial communities, explaining 27% and 21%, respectively (*p* < 0.001; Table S4). The Venn diagram revealed that 56.7% of bacterial zOTUs were shared between all plastisphere and soil samples (Fig. S7).

### Network analysis and bacterial assembly based on the microcosm experiment 2

**Fig. 3:**
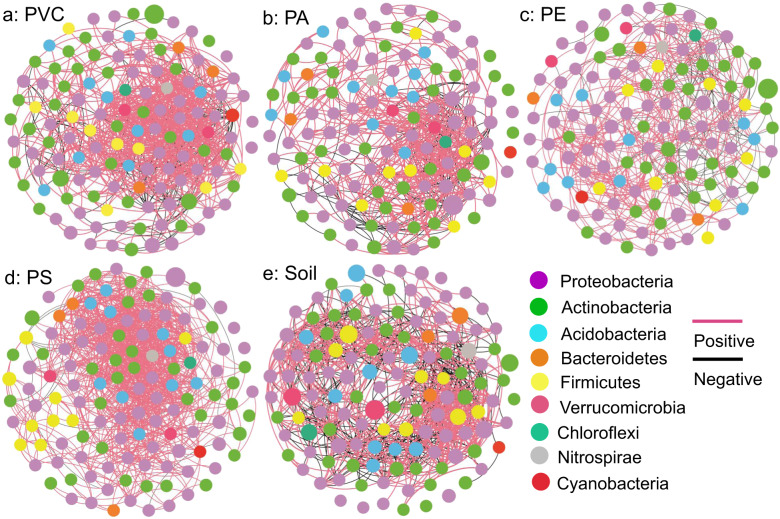
Relationships between prevalent bacterial zOTUs. Co-occurrence networks of different plastisphere (**a**, **b**, **c**, and **d**) and soil (**e**) bacterial zOTUs. The size of the circle indicated the relative abundance of the zOTUs.

Relationships between prevalent bacterial zOTUs (present in at least 80% samples) were revealed by the co-occurrence network analysis (Fig. 3 and Table S5). More positive than negative edges were detected in co-occurrence networks of the plastisphere. Fewer negative edges were found in co-occurrence networks of plastisphere (PVC: 116 (13%), PA: 127 (20%), PE: 111 (20%), and PS: 19 (2%)) compared to the soil (373 (40%)). The modularity of plastisphere co-occurrence networks (0.32–0.76) was lower than that of the soil (1.68). The highest average degree (16.7) and most positive edges (1034 (98%)) were observed in the co-occurrence network of PS plastisphere. The co-occurrence network of PE plastisphere had the fewest edges (557) and lowest density (0.07).

**Fig. 4:**
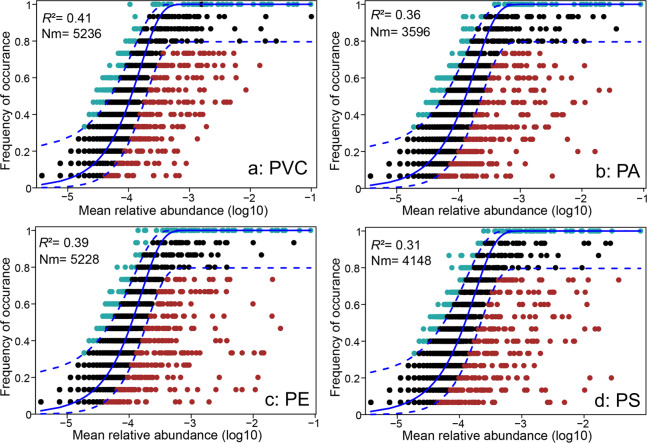
Fit of neutral model for plastisphere. Each point represented a bacterial zOTUs, and different colors indicated zOTUs that occur more or less frequently than predicted by neutral model. The predicted occurrence frequency was shown as a solid blue line and dashed lines indicated the 95% confidence interval around the neutral model. The *R*^2^ indicated the fit to neutral model, and Nm indicated the metacommunity size times immigration.

The neutral community model was used to assess the contribution of stochastic process to plastisphere microbial community assembly (Fig. 4). For all MP types, AIC scores of the neutral model prediction were lower than the binomial distribution (Fig. S8a). Principal coordinates analysis showed that plastisphere samples were distinctly clustered based on zOTUs in the above and below- partitions of the neutral model (*p* = 0.028; Fig. 4 and S8b). A majority of the distribution of zOTUs (~89%) in the plastisphere could be predicted by the neutral community model (Fig. 4 and S9). Compared with other phyla, zOTUs belonging to phylum Patescibacteria had the highest ratio (34%) falling below the neutral model prediction (Fig. S9). The lowest value of Nm was found in the neutral model of PA compared to other MPs (Fig. 4).

### Functional prediction and potential bacterial pathogens based on microcosm experiment 2

**Fig. 5:**
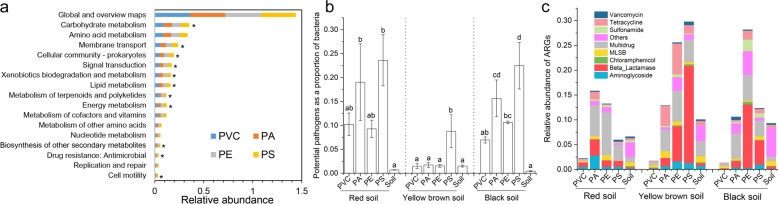
Functional prediction, potential pathogen and ARGs. **a** Metabolic pathways of bacterial communities in different plastispheres predicted by Tax4Fun2. The asterisk indicated significant difference between different plastispheres (significant level: *p* < 0.05). **b** Ratio of potential pathogens/bacteria (mean ± SE; *n* = 5) detected in different plastisphere and soil samples. The different letter indicated significant difference between different samples from the same soil environment. **c** Relative abundance of ARGs (mean; *n* = 5) in different plastisphere and soil samples.

Functional profiles of the Tax4Fun2 prediction were summarized based on KEGG pathways, and the biological metabolic pathways were divided into three levels (level 1, level 2, and level 3). Our results showed that 356 metabolic pathways at level 1 presented in plastisphere bacterial communities. At level 3, metabolism, cellular processes, genetic information processing, environmental information processing and human diseases were primary metabolic pathways. At level 2, the metabolic pathway of drug resistance: antimicrobial (abundance: 0.01) including beta-Lactam resistance was found in all plastispheres, and the global and overview maps (abundance: 0.36) had the highest abundance in all predicted functional genes (Fig. 5a). Biosynthesis of antibiotics (abundance: 0.05) as a subsystem was included in the global and overview maps. A large number of primary metabolic pathways (11/17) significantly differed between different plastispheres (*p* < 0.05; Fig. 5a).

Overall, the ratio of potential bacterial pathogens to overall bacteria in the plastisphere was 12.4 times higher than that in the soil bacterial community (*p* < 0.001; Fig. S10). A total of 73 potential bacterial pathogens were detected in all plastispheres. Apart from the Yellow brown soil, a higher ratio of potential bacterial pathogens/bacteria was observed in each MPs plastisphere compared to the soil (*p* < 0.05; Fig. 5b). In the Yellow brown soil, a similar ratio of potential bacterial pathogens/bacteria was found among PVC, PA, PE, and soil, but this ratio was significantly higher in the PS (*p* < 0.05; Fig. 5b). The composition of dominant potential bacterial pathogenic species (abundance >1%) in the plastisphere was different from the soil (Fig. S11). *Bordetella avium* (34%) and *Pseudomonas fluorescens* (13%) predominated in the detected potential pathogenic species of plastisphere, following by *Pseudomonas protegens* (6.8%), *Burkholderia* sp. (5.2%) and *Pseudomonas syringae* (5.1%).

### Characterization of the plastisphere antibiotic resistome based on microcosm experiment 2

A total of 102 ARGs and 3 MGEs were detected in all plastisphere samples, in 9 ARGs types (Fig. 5c). Relative abundance of plastisphere ARGs varied across soil and MPs types (Fig. 5c). PERMANOVA tests further showed that MP types had a significant influence on the change of plastisphere ARGs, explaining 18% of the variation (*p* < 0.001; Table S4). Apart from the PVC, a higher relative abundance of ARGs and MGEs was overall observed in plastisphere compared to soil (Fig. 5c and Fig. S12). Especially for PE, in three different soil environments, relative abundance of ARGs was all significantly higher than that in the soil (*p* < 0.05; Fig. 5c). The Beta_Lactamase and Multidrug were two dominant ARGs types in the PE, PA and PS plastispheres, and their relative abundances were significantly higher than those in the soil (*p* < 0.05; Fig. 5c). Principal coordinates analysis of ARGs showed that most soil samples were clustered and distinctly separated from plastisphere samples (Fig. S13). The ARG profiles of samples significantly differed between treatments (PERMANOVA, *p* < 0.001; Fig. S13). Most ARGs (71%) were shared between plastisphere and soil, and only 11% of ARGs was unique in the plastisphere (Fig. S14). For the same MPs, only 45–51% of ARGs were shared in the plastisphere between different soil environments (Fig. S15).

### The relationship between bacterial communities and antibiotic resistance genes based on microcosm experiment 2

**Fig. 6:**
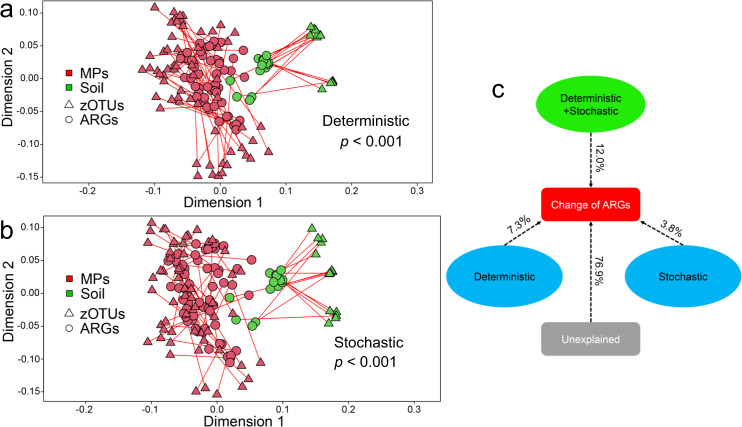
The contribution of deterministic and stochastic processes acting on bacterial communities to the change of ARGs in the plastisphere. Procrustes test revealed the significant correlation between ARG profiles and bacterial communities from deterministic process (**a**) and stochastic process (**b**), respectively. **c** Partial redundancy analysis differentiated effects of deterministic process and stochastic process on the variations in ARGs.

The co-occurrence network of ARGs and bacterial communities is presented in Fig. S16. Three ARGs (*fox5*, *aadA1*, and *aadA2*) all had significant positive correlations with multiple bacterial phyla (*p* < 0.05). Three bacterial phyla (Acidobacteria, Rokubacteria, and Entotheonellaeota) were significantly positively correlated with at least two ARGs, respectively (*p* < 0.05). Procrustes tests revealed a significant correlation between ARG profiles and deterministic and stochastic processes acting on bacterial communities, respectively (*p* < 0.001; Fig. 6a and b). Partial redundancy analysis further showed that deterministic process acting on bacterial community could explain 7.32% of ARGs variation, more than 3.84% of stochastic process (Fig. 6c).

### Effects of manure, temperature and moisture on the microbiome and antibiotic resistome in the plastisphere based on microcosm experiment 3

**Fig. 7:**
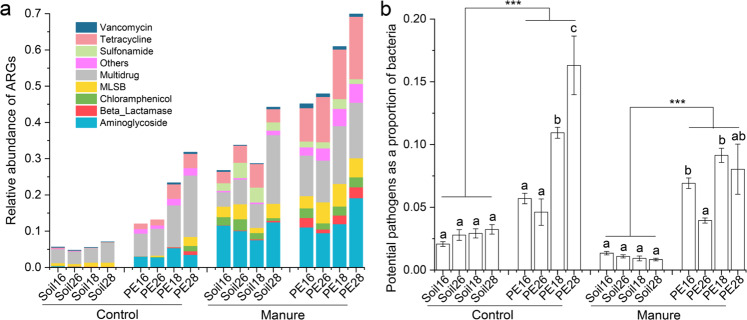
Effects of manure, temperature and moisture on ARGs and potential pathogen. **a** Relative abundance of ARGs (mean; *n* = 5) in the plastisphere and soil sample from different treatments. **b** Ratio of potential pathogens/bacteria (mean ± SE; *n* = 5) detected in the plastisphere and soil sample from different treatments. The different letter indicated significant difference between different samples (significant level: *p* < 0.05), and the “***” indicated *p* < 0.001.

In microcosm experiment 3, we obtained 3,613,142 sequences and 9116 bacterial zOTUs at 100% identity. The bacterial zOTUs table was rarefied to 19,783 reads per sample. A total of 127 ARGs were detected in all soil and plastisphere samples, in 9 ARGs types (Fig. 7a). The addition of manure significantly increased the relative abundance of ARGs in the plastisphere (by 179%) and soil (by 480%) samples compared to the control (*t* test, *p* <0.001; Fig. 7a). The addition of manure did not overall alter the ratio of potential pathogens/bacteria in the plastisphere samples (*t* test, *p* = 0.10; Fig. 7b). However, in the control and manure treatments, the ratio of potential pathogens/bacteria in the plastisphere samples was 3.4 and 6.7 times higher than that in soil samples, respectively (*t* test, *p* < 0.001; Fig. 7b). The PCoA of bacterial communities and ARG profiles all revealed that samples were clustered based on whether or not manure was added (Figs. S17 and S18). The PERMANOVA further indicated that manure could explain 13% of the variation in the bacterial communities and 57% of the variation in the ARG profiles (*p* < 0.001; Table S6). Temperature and moisture all had a distinct influence on the structure of bacterial community and ARG profiles in the plastisphere samples (PERMANOVA, *p* < 0.05; Table S6 and Figs. S17 and S18), whereas, effects of moisture (explaining 15% of the variation in the bacterial communities and 12% of the variation in the ARG profiles) were greater than temperature (explaining 7% and 2%). The elevation of soil temperature and moisture all overall enhanced the relative abundance of ARGs in the plastisphere samples (Fig. 7a), and the interaction between temperature and moisture was significant (PERMANOVA, *p* < 0.05; Table S6). Overall, the elevation of soil moisture increased the ratio of potential pathogens/bacteria in the plastisphere samples (Fig. 7b). In the treatment without adding manure, a significantly higher ratio of potential pathogens/bacteria in the plastisphere samples was observed in the 80% moisture of soil environment compared to the 60% treatment, and the elevation of soil temperature distinctly increased this effect of moisture (ANOVA, *p* < 0.05; Fig. 7b).

### Metagenomic insights into the composition, function, and antibiotic resistome of microbial communities in different plastispheres based on the field experiment

**Fig. 8:**
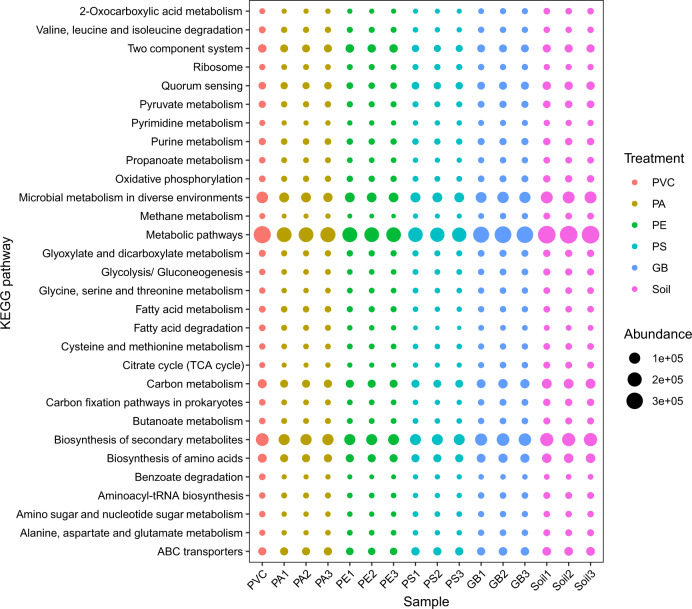
Bubble Plot revealing profiles of the 30 most abundant KEGG pathways from different substrates (PVC, PA, PE, PS, GB, and soil). The abundance of KEGG pathway was represented as RPKM. The profiles were prepared by assembling the identified KOs into broader functional categories at different functional levels. PVC polyvinyl chloride, PA polyamide, PE polyethylene, PS polystyrene, and GB glass bead.

Across 16 metagenomic sequencing of samples, a total of 199 G raw data were obtained with minimum 10 G raw data per sample. The 1,232,276,084 clean reads were determined, and 9,222,307 contigs were identified with minimum 110,018 contigs per sample. The composition of microbial communities in the plastisphere was obviously different from those in glass beads and soil samples at the phylum, family and species levels (Figs. S19 and S20). For example, a higher proportion of Acidobacteria was found in glass bead (8.4%) and soil (23%) samples compared to plastispheres (0.9%), and plastispheres hosted higher abundance of Caulobacteraceae and *Ferrovibrio terrae*. The PCoA of microbial communities and functions all indicated that samples were clustered based on different substrates, and plastisphere samples were distinctly separate from bead glass and soil samples (PERMANOVA, *p* < 0.05; Fig. S21). We further found that the structure of microbial community (defined by the PCo1 axis) was strong correlation with substrate hydrophobicity (Spearman, *R*^2^ = 0.78, *p* < 0.001; Fig. S22). By the functional prediction of KEGG, a variety of pathways were observed in the plastisphere, including Metabolic pathways, Biosynthesis of secondary metabolites, Carbon metabolism, ABC transporters, and so forth (Fig. 8). Compared with glass bead and soil samples, two metabolic pathways (Xenobiotics biodegradation and metabolism and drug resistance: antimicrobial) were enriched in the plastisphere, especially for PE (*p* < 0.05; Fig. S23). Abundant KOs belonging to Carbon, Nitrogen, and Sulfur metabolisms were detected in plastispheres (Figs. S24–26), and plastispheres significantly enhanced the abundance of two KOs belonging to arsenic resistance (*p* < 0.05; Fig. S27). In the annotation of CAZyme, higher abundance of Carbohydrate Esterases was observed in plastispheres compared to bead glass and soil samples (*p* < 0.05; Fig. S28). Metagenomic analysis showed that the abundance of ARGs in the PE plastisphere was significantly higher than that in other samples (*p* < 0.05; Fig. S29). Microbes that perform the same function in plastispheres were different from bead glass and soil samples (Figs. S30 and S31). *Pseudomonas* had an important contribution to the variation of fluoroquinolone, chloramphenicol and beta_lactam resistance genes in plastispheres (Fig. S31).

## Discussion

Our study found that the alpha diversity of plastisphere microbial communities was distinctly lower than the source community (soil), structures of MPs-associated communities diverged from the glass beads and soil, and variations in the structure of plastisphere communities were strongly linked to the substrate hydrophobicity, which suggested the selection of MPs (filtering) for microbiota in the plastisphere. This has also been demonstrated in a previous study in aquatic ecosystems [35]. Our study further revealed that MP types have an important effect on the change of the microbial community in the plastisphere, indicating that different plastic types exert a distinct selectivity on the microbial community. Some studies of aquatic ecosystems also support this point [19, 65, 66]. One possible reason is that different types of MPs may provide distinct niches for microbiota due to differences in plastic properties (e.g., surface area as a colonization site and hydrophobicity) [67]. Our study further found that different size of PE MPs had a distinct bacterial community in the plastisphere, which suggested that MPs surface area has a significant effect on the colonization of microbiota. In addition, our results also shown that plastic hydrophobicity has an important contribution to the colonization of microbiota, which was consistent with the result of a previous study [35].

The results of network analysis indicated that more negative correlations between bacterial zOTUs were detected in the soil compared to the plastisphere, suggesting that competition within plastisphere bacterial communities may be weaker than that in the soil. Compared with the soil, limited resources in the plastisphere may contribute to this difference. Since the degradation of MPs is difficult, microbes may tend to cooperate to make use of them. This fits quite well to the stress gradient hypothesis that positive interactions tend to dominate over competitive interactions in “harsher” environments, as found in plants and the compost community of copper stress [68, 69]. We further found that the relationship between plastisphere bacterial zOTUs was predominantly positive in the soil ecosystem. Positive connection commonly could be explained by two alternative reasons: niche overlap [70] (between phylogenetically related taxa) and mutualistic/facilitative interactions [71] (usually between distinct bacterial taxa). In our study, many zOTUs belonging to distinct phyla co-occurred in the network, which supported the assumption of positive interactions to some extent. Our study used the neutral model to better understand the assembly of the plastisphere bacterial community in the soil ecosystem. The distribution of most of bacterial zOTUs was successfully predicted by neutral processes in our study, indicating that the distribution of these zOTUs in the plastisphere is independent of bacterial functional traits (e.g., niche) and mainly shaped by passive dispersal and ecological drift [72]. In addition, we also identified some bacterial taxa which were not predicted by neutral processes, supporting the idea that MPs can act as an environmental filter to bacteria.

Results of functional prediction from amplicon and metagenomics sequencings all revealed that diverse metabolic pathways exist in the plastisphere community, including numerous carbon, nitrogen, and sulfur metabolic pathways. We further found that the profiles of microbial functions were different between plastisphere and soil. Recently, a study also showed that microbial turnover and nutrient efficiency was enhanced in the plastisphere [21]. These all suggested that plastisphere communities could participate in a large number of important ecological processes in the soil ecosystem. In addition, in our study, many metabolic pathways belonging to xenobiotics biodegradation and metabolism and arsenic resistance were enriched in the plastisphere, indicating that plastisphere communities might be suffering from more environmental stress compared to the soil. Similar results were also reported from aquatic ecosystems [19, 73]. Multiple metabolic pathways involving in antibiotic resistance were also detected in the present study, especially for PE plastisphere, and they were the primary metabolic pathway in the plastisphere community. This suggested that the plastisphere is a “hot spot” of ARGs, which supported the result that many unique ARGs were observed in the plastisphere. This may be because MPs can absorb organic compounds (including antibiotics) when in the soil due to their hydrophobicity, which produces a pressure on bacteria [20, 29, 74, 75]. We also found that apart from the PVC, the relative abundance of MGEs in the plastisphere was significantly higher than that in the soil. A previous study has indicated exchange of genes in the plastisphere via horizontal gene transfer [20]. Therefore, exchange of ARGs may be frequently occurring in some plastispheres from the soil ecosystem via MGEs.

Our study found the presence of a range of potential pathogens and ARGs in the plastisphere, suggesting that there is a high risk of ARGs entering potential pathogens via MGEs in the plastisphere. Several recent studies also detected multiple ARGs in the plastisphere [29, 30]. We further identified that the ratio of potential pathogens/bacteria and abundance of ARGs in the plastisphere were overall clearly higher than those in the soil. This provided solid evidence, similar to that from aquatic ecosystems [26, 76], that MPs also act as a reservoir and refuge for ARGs and potential pathogens in the soil. In our study, abundant multidrug resistance genes were detected in the plastisphere. One possible reason is that microbiota are subjected to a variety of stresses in the plastisphere due to MPs commonly absorbing multiple pollutants [15, 77]. This point was also partly supported by the results of metagenomic sequencing that many metabolic pathways belonging to pollutant were enriched in the plastisphere. It is well established that Multidrug genes could provide resistance to multiple antibiotics, and thus carry a high risk for ecosystems [78]. These findings all indicated that MPs as a vector may accelerate the dispersal of ARGs in the ecosystem. However, ARGs in the plastisphere varied across different types of MPs. In our study, the enrichment of ARGs was not observed in the PVC plastisphere. Different types of MPs present different properties at their surface [67], which may affect the selection of ARGs in the plastisphere. That surface properties of MPs significantly affected the absorption of antibiotics has been reported [79, 80]. Therefore, the assessment of MP risk for the spread of ARGs requires a deeper understanding of MPs types in future work.

As we hypothesized, the present study revealed that the contribution of deterministic process acting on bacterial communities to the antibiotic resistome of the plastisphere is higher than that of stochastic processes. This indicated that the selection of MPs for some bacteria may play an important role in the enrichment of ARGs in the plastisphere. Although previous studies have shown a significant correlation between bacterial community structure and ARG profiles [33, 43, 50], our findings highlight the significance of bacterial deterministic process in the variation of ARGs in the plastisphere. Our study further identified three bacterial phyla that were positively correlated with at least two ARGs by network analysis. The results of metagenomic sequencing also confirmed that *Pseudomonas* (commonly potential bacterial pathogen [81]) had an important contribution to the variation of fluoroquinolone, chloramphenicol and beta_lactam resistance genes in plastispheres. In the present study, the metabolic pathway of beta-Lactam resistance and abundant Beta_Lactamase ARGs co-occur in the plastisphere. All this supports the view that some bacteria selected by MPs contributed to the enrichment of ARGs in the plastisphere.

We found that, similarly to the rhizosphere and phyllosphere [44, 45], the addition of manure significantly increased ARGs in the PE plastisphere. This suggested that the ARGs introduced by human activities will accumulate in the plastisphere, especially in soils with long-term manure application. At the same time, our results revealed that the elevation of soil temperature and moisture all distinctly enhanced ARGs of plastispheres. This indicated that the warm and humid environment is favorable for the increase of ARGs in the plastisphere, which may be related to variations in microbial communities. Previous studies have shown that microbiota are sensitive to changes in temperature and moisture [37, 38], and microbial community commonly contributes to changes in the antibiotic resistome in multiple environments [33, 43, 50]. We further employed Procrustes test and Mantel analysis to also reveal that there is a significant correlation between microbes and ARGs in the plastisphere (*p* < 0.05). Therefore, in a scenario of global change, plastisphere-mediated dispersal of ARGs needs more attention in the soil ecosystem.

In conclusion, we here revealed the assembly of microbial communities and the antibiotic resistome of different types of plastispheres from diverse soil environments. The MPs select for microbial communities in the plastisphere, and these microbiota are involved in a variety of ecological processes in the soil ecosystem. Plastispheres act as a reservoir and refuge for ARGs and potential pathogens in the soil, especially in a scenario of the application of manure or temperature and moisture increase. Moreover, plastisphere represents a hotspot of increased potential for horizontal gene transfer, which may accelerate the spread of antimicrobial resistance globally. Although neutral processes dominate community assembly in the plastisphere, the selection of MPs for some bacteria may play an important role in the enrichment of ARGs. These findings extend our knowledge on the microbial community, function and antibiotic resistome of the plastisphere in the soil under global change, highlighting that community ecology can be useful for explaining variations in ARGs in the plastisphere.

## Supplementary information

Supplementary information

## Data Availability

We submitted the obtained raw sequencing data to the NCBI Sequence Read Archive under the accession number PRJNA397169. The authors declare that the other main data supporting the findings of this study are available within this Article and in the Supplementary Information files. Extra data supporting the findings of this study are available from the corresponding author upon reasonable request.

## Code availability

Custom codes for all analyses are available from the corresponding authors upon request.
