# Correction: Soil plastispheres as hotspots of antibiotic resistance genes and potential pathogens

**DOI:** 10.1038/s41396-021-01137-z

**Published:** 2021-10-27

**Authors:** Dong Zhu, Jun Ma, Gang Li, Matthias C. Rillig, Yong-Guan Zhu

**Affiliations:** 1 State Key Laboratory of Urban and Regional Ecology, Research Center for Eco-Environmental Sciences, Chinese Academy of Sciences, Beijing, China; 2 Key Laboratory of Urban Environment and Health, Institute of Urban Environment, Chinese Academy of Sciences, Xiamen, China; 3 Freie Universität Berlin, Institute of Biology, Berlin, Germany; 4 Berlin-Brandenburg Institute of Advanced Biodiversity Research, Berlin, Germany

**Keywords:** Microbial ecology, Environmental sciences

Correction to: *The ISME Journal* 10.1038/s41396-021-01103-9, published online 28 August 2021

The article “Soil plastispheres as hotpots of antibiotic resistance genes and potential pathogens”, written by Dong Zhu, Jun Ma, Gang Li, Matthias C. Rillig and Yong-Guan Zhu, was originally published electronically on the publisher’s internet portal on 28 August 2021 without open access. With the author(s)’ decision to opt for Open Choice the copyright of the article changed on 13 September 2021 to © The Author(s) 2021 and the article is forthwith distributed under a Creative Commons Attribution 4.0 International License, which permits use, sharing, adaptation, distribution and reproduction in any medium or format, as long as you give appropriate credit to the original author(s) and the source, provide a link to the Creative Commons licence, and indicate if changes were made.

The images or other third party material in this article are included in the article’s Creative Commons licence, unless indicated otherwise in a credit line to the material. If material is not included in the article’s Creative Commons licence and your intended use is not permitted by statutory regulation or exceeds the permitted use, you will need to obtain permission directly from the copyright holder.

To view a copy of this licence, visit http://creativecommons.org/licenses/by/4.0/.

The original article has been corrected.

