# Supplementary material for: Soil plastispheres as hotspots of antibiotic resistance genes and potential pathogens
Source: ISME J. 2021 Aug 28;16(2):521–32. doi: 10.1038/s41396-021-01103-9 (PMC8776808; doi:10.1038/s41396-021-01103-9)
Supplement: Supplementary file 1 — Supplementary information [file 41396_2021_1103_MOESM1_ESM.docx]

**Supporting Information for**

**Soil plastispheres as hotpots of antibiotic resistance genes and potential pathogens**

Dong Zhu, ^a^ Jun Ma, ^b^ Gang Li, ^b^ Matthias C. Rillig, ^c, d^ Yong-Guan Zhu ^*, a, b^

^a^ State Key Laboratory of Urban and Regional Ecology, Research Center for Eco-Environmental Sciences, Chinese Academy of Sciences, Beijing 100085, China.

^b^ Key Laboratory of Urban Environment and Health, Institute of Urban Environment, Chinese Academy of Sciences, 1799 Jimei Road, Xiamen 361021, China.

^c^ Freie Universität Berlin, Institute of Biology, 14195 Berlin, Germany.

^d^ Berlin-Brandenburg Institute of Advanced Biodiversity Research, 14195 Berlin, Germany.

* Correspondence to: Yong-Guan Zhu Email: ygzhu@rcees.ac.cn

**This PDF file includes:**

Tables S1 to S6

Figures S1 to S31

**Table S1** Soil basic properties.

| Type | Landuse | Clay (%) | pH | EC (us cm^-1^) | C (%) | N (%) | NH_4_-N (mg kg^-1^) | NO_3_-N (mg kg^-1^) |
| --- | --- | --- | --- | --- | --- | --- | --- | --- |
| Red soil | Arable | 1.35 | 6.68 | 621.60 | 3.74 | 0.37 | 12.12 | 145.25 |
| Yellow Brown soil | Arable | 1.56 | 6.41 | 110.30 | 2.23 | 0.15 | 7.87 | 5.49 |
| Black soil | Arable | 0.97 | 6.47 | 156.10 | 6.59 | 0.14 | 10.17 | 23.00 |
| Black soil | Forest | 1.13 | 6.96 | 374.90 | 8.56 | 0.18 | 12.12 | 63.76 |

**Table S2** The specific surface areas and average adsorption pore widths of the four MPs.

| Properties | PVC | PA | PE | PS |
| --- | --- | --- | --- | --- |
| Specific surface areas | 3.70 | 0.63 | 1.66 | 2.16 |
| Average adsorption pore widths | 6.76 | 0.54 | 4.79 | 7.02 |

**Table S3** The hydrophobicity (contact angle) of substrate used in this study.

| Particle type | Water substrate contact angle (degrees) | Reference for contact angle |
| --- | --- | --- |
| Polyvinyl chloride | 83 | Määttä et al., 2007 [1] |
| Polyamide | 77.1 | Bismarck et al., 1999 [2] |
| Polyethylene | 101.7 | De Geyter et al., 2008 [3] |
| Polystyrene | 87 | Li et al., 2007 [4] |
| Glass bead | 51 | Nowak et al., 2013 [5] |

1. Määttä J, Koponen HK, Kuisma R, Kymäläinen HR, Pesonen-Leinonen E, Uusi-Rauva A, et al. Effect of plasticizer and surface topography on the cleanability of plasticized PVC materials*.* Appl Surf Sci. 2007;253:5003-10.

2. Bismarck A, Richter D, Wuertz C, Springer J. Basic and acidic surface oxides on carbon fiber and their influence on the expected adhesion to polyamide*.* Colloid Surface A. 1999;159:341-50.

3. De Geyter N, Morent R, Leys C. Surface characterization of plasma-modified polyethylene by contact angle experiments and ATR-FTIR spectroscopy*.* Surfaces. 2008;40:608-11.

4. Li Y, Pham JQ, Johnston KP, Green PF. Contact angle of water on polystyrene thin films:Effects of CO_2_ environment and film thickness*.* Langmuir. 2007;23:9785-93.

5. Nowak E, Robbins P, Combes G, Stitt EH, Pacek AW. Measurements of contact angle between fine, non-porous particles with varying hydrophobicity and water and non-polar liquids of different viscosities*.* Powder Technol. 2013;250:21-32.

**Table S4** The effects of MPs and soil types on the differentiations of plastisphere bacterial communities and antibiotic resistance genes (ARGs) profile based on the PERMANOVA test.

| Response variable |  | MPs | Soil | MPs×Soil |
| --- | --- | --- | --- | --- |
| Bacterial community | F | 11.3 | 13.4 | 2.81 |
|  | *R*^2^ | 0.27 | 0.21 | 0.13 |
|  | *p* | < 0.001 | < 0.001 | < 0.001 |
| ARGs profile | F | 4.28 | 1.36 | 1.43 |
|  | *R*^2^ | 0.18 | 0.04 | 0.12 |
|  | *p* | < 0.001 | 0.078 | 0.005 |

**Table S5** Bacterial co-occurrence network characteristics.

|  | Node | Positive edge | Negative edge | Average degree | Modularity | Density | Average clustering coefficient |
| --- | --- | --- | --- | --- | --- | --- | --- |
| PVC | 126 | 810 | 116 | 14.70 | 0.39 | 0.12 | 0.52 |
| PA | 126 | 504 | 127 | 10.02 | 0.64 | 0.08 | 0.46 |
| PE | 126 | 446 | 111 | 8.84 | 0.76 | 0.07 | 0.42 |
| PS | 126 | 1034 | 19 | 16.71 | 0.32 | 0.13 | 0.51 |
| Soil | 126 | 559 | 373 | 14.79 | 1.68 | 0.12 | 0.52 |

| Response variable |  | Man. | Tem. | Moi. | Man.×Tem. | Man.×Moi. | Tem.×Moi. | Man.×Tem. ×Moi. |
| --- | --- | --- | --- | --- | --- | --- | --- | --- |
| Bacterial community | F | 8.93 | 4.78 | 10.1 | 1.81 | 5.30 | 2.37 | 2.20 |
|  | *R*^2^ | 0.13 | 0.07 | 0.15 | 0.03 | 0.08 | 0.04 | 0.03 |
|  | *p* | < 0.001 | < 0.001 | < 0.001 | 0.049 | < 0.001 | 0.013 | 0.016 |
| ARGs profile | F | 116 | 4.19 | 24.1 | 7.70 | 7.41 | 6.96 | 4.47 |
|  | *R*^2^ | 0.57 | 0.02 | 0.12 | 0.04 | 0.04 | 0.03 | 0.02 |
|  | *p* | < 0.001 | 0.014 | < 0.001 | 0.002 | 0.001 | 0.001 | 0.019 |

**Table S6** The effects of MPs and soil types on the differentiations of plastisphere bacterial and archaeal communities and antibiotic resistance genes (ARGs) profile based on the PERMANOVA test.


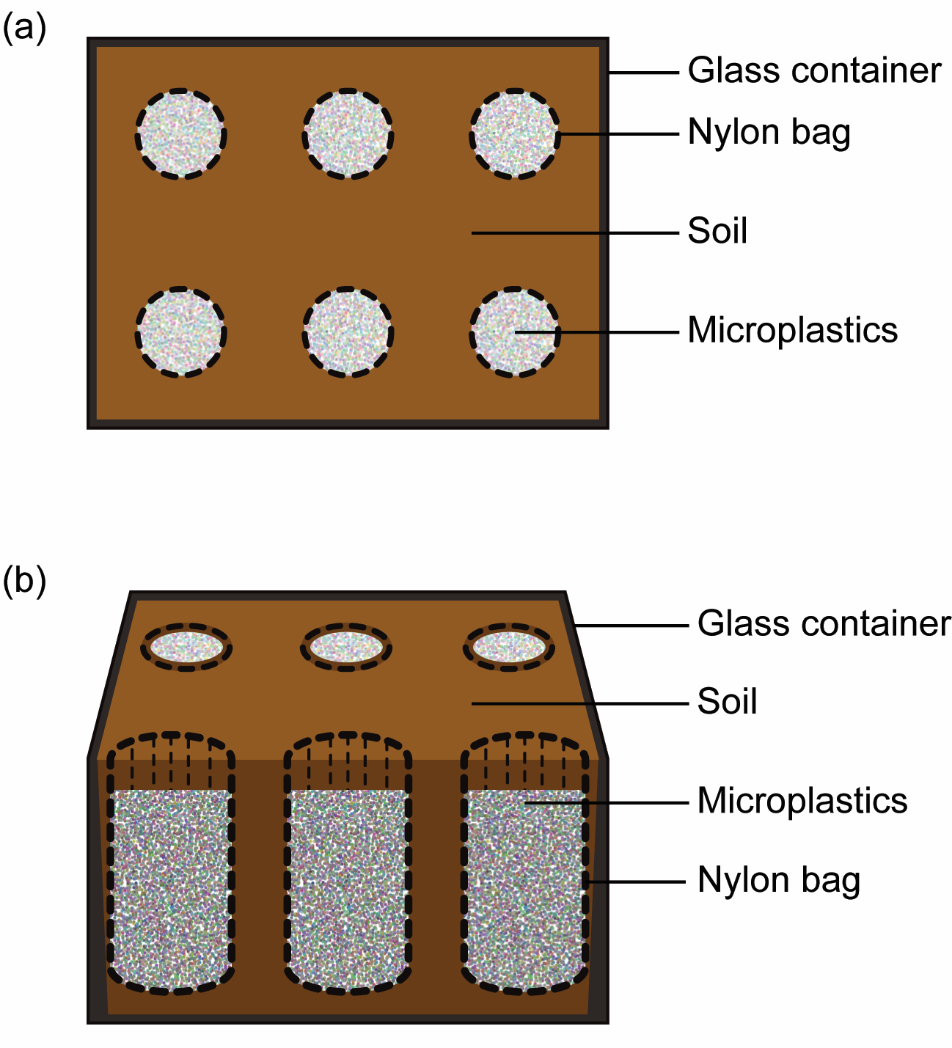


**Figure S1.** Schematic diagram of microplastics incubation.


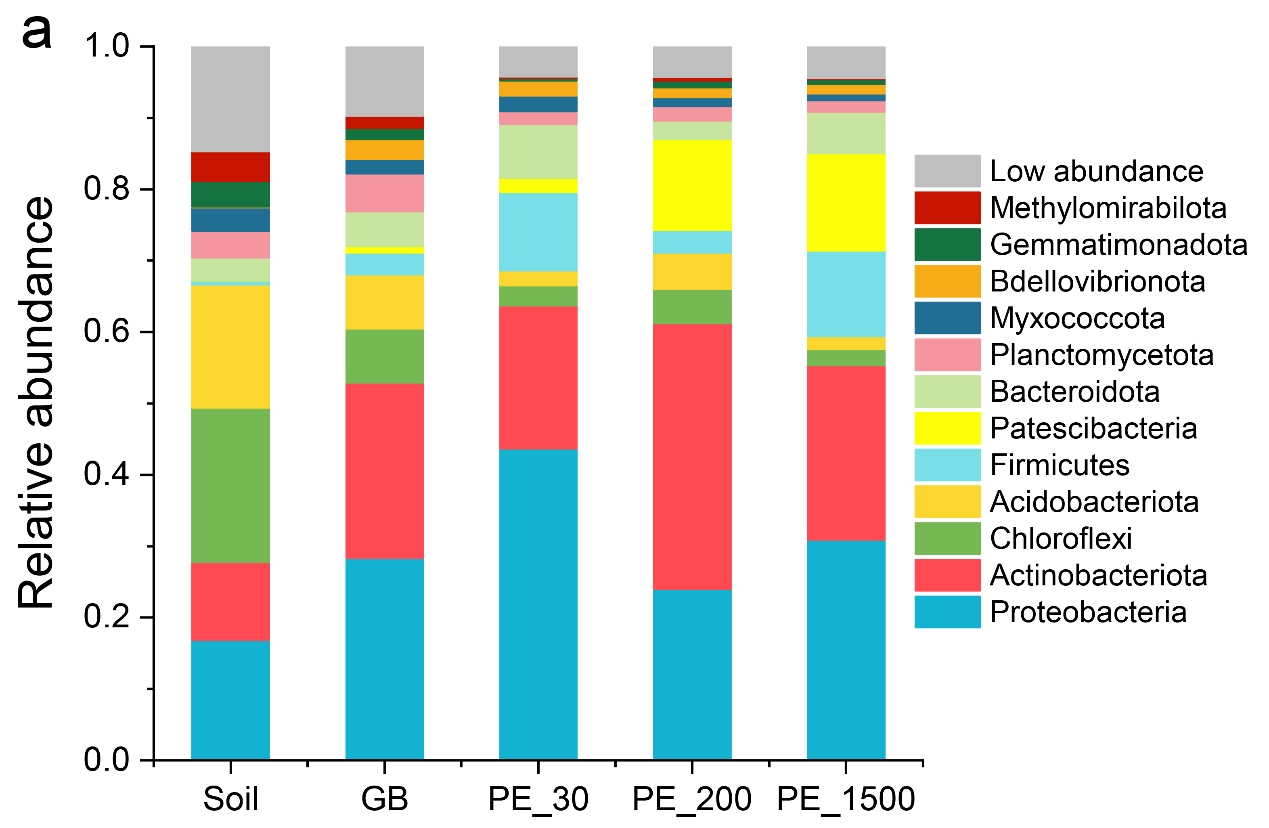

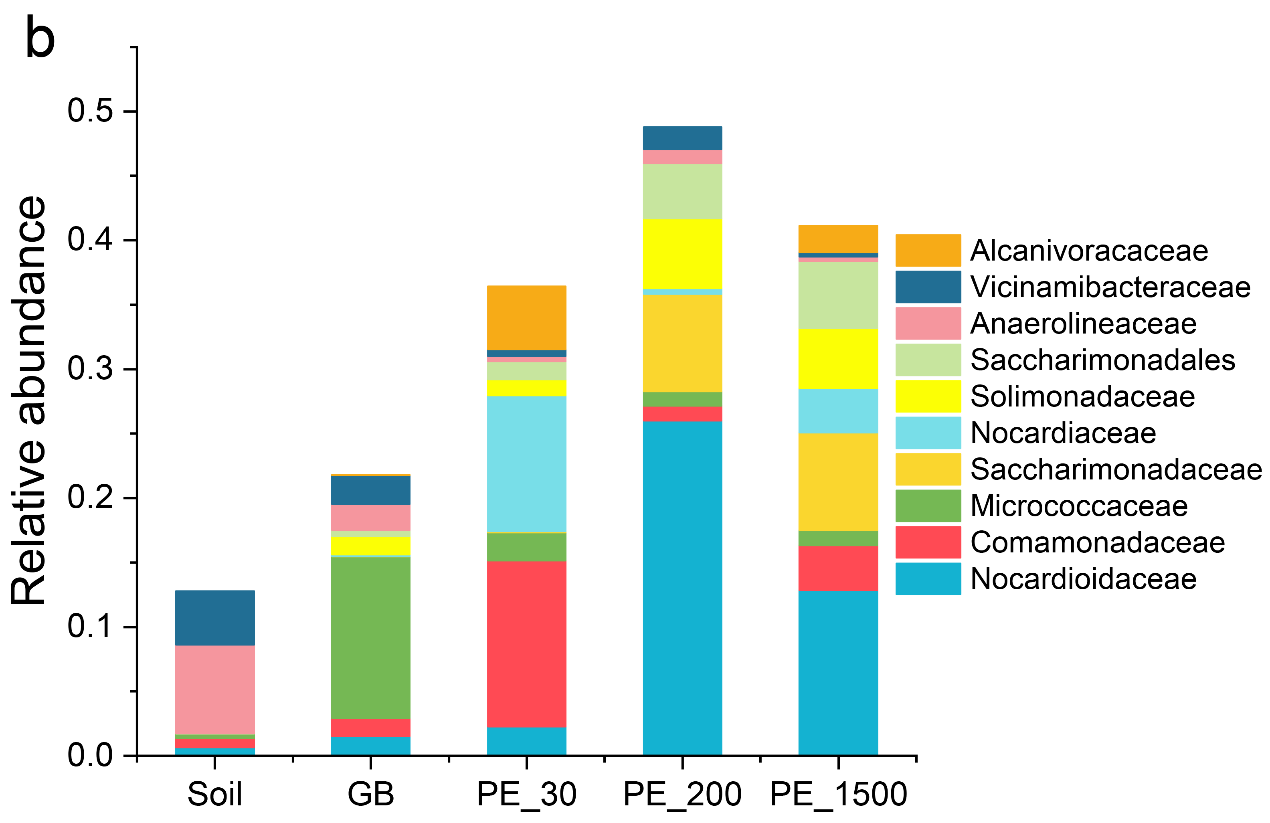


**Figure S2.** The composition of bacterial communities (Phylum level: a; Family level: b) in different size of PE plastispheres (30, 200 and 1500 μm), 30 μm glass bead (GB) and soil samples. For the phylum level, the relative abundance > 1% of phyla were presented, and, for the family level, the 10 most abundant families were showed.


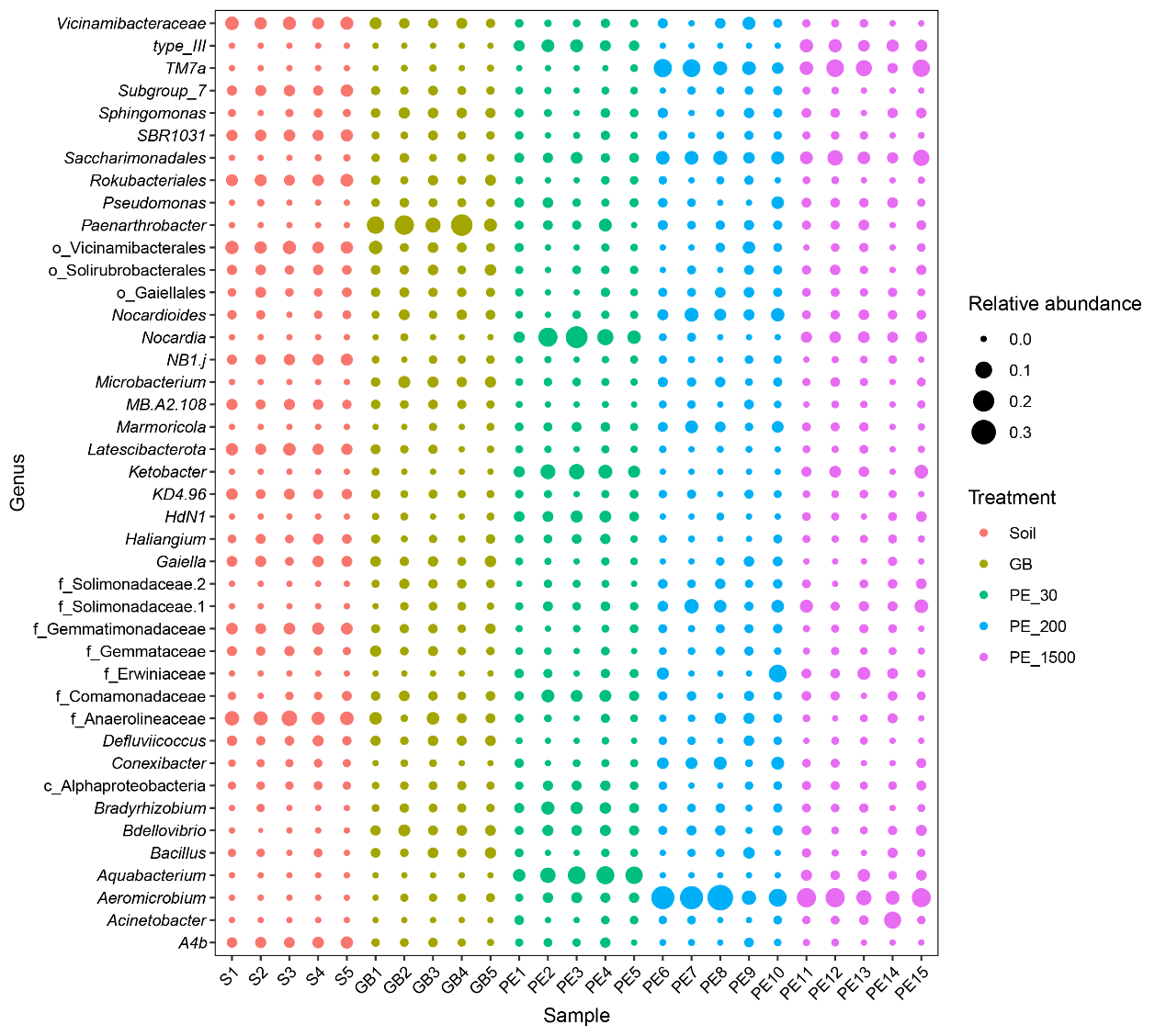


**Figure S3.** Bubble Plot revealing the composition of bacterial communities (Genus level: the 30 most abundant genera) in different size of PE plastispheres (30, 200 and 1500 μm), 30 μm glass bead (GB) and soil samples.


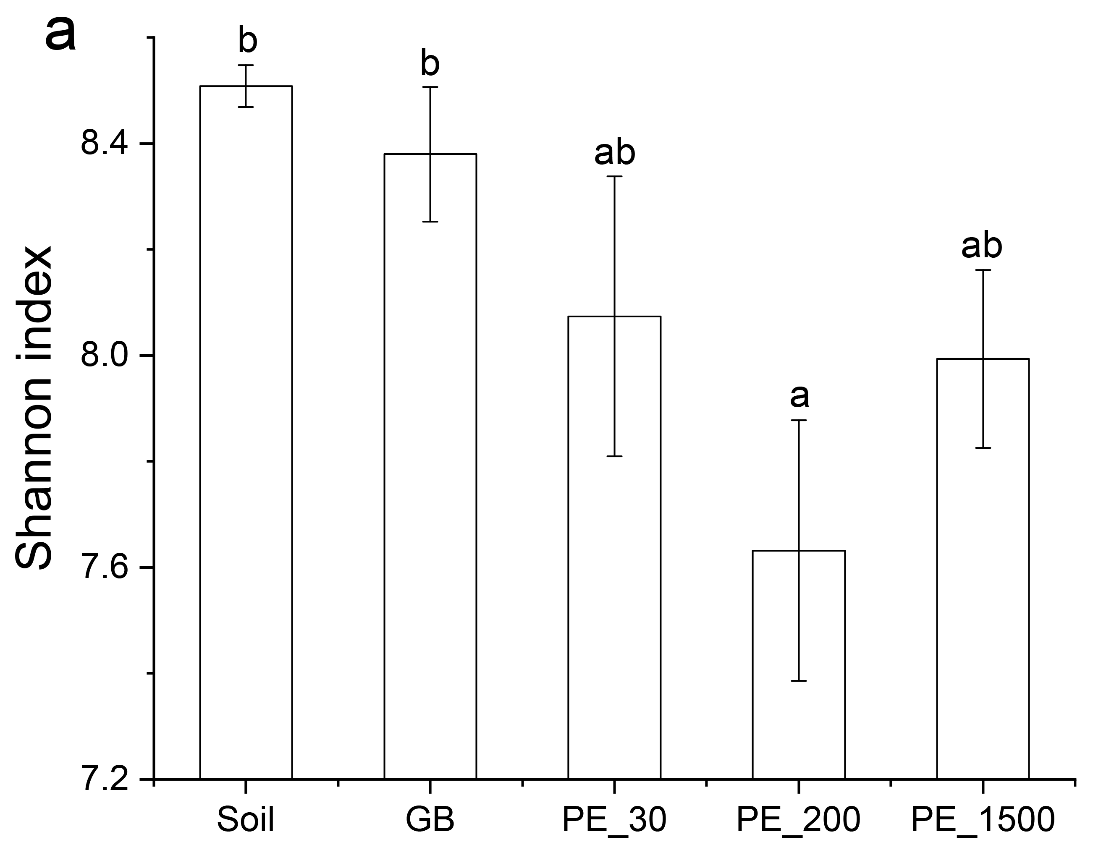

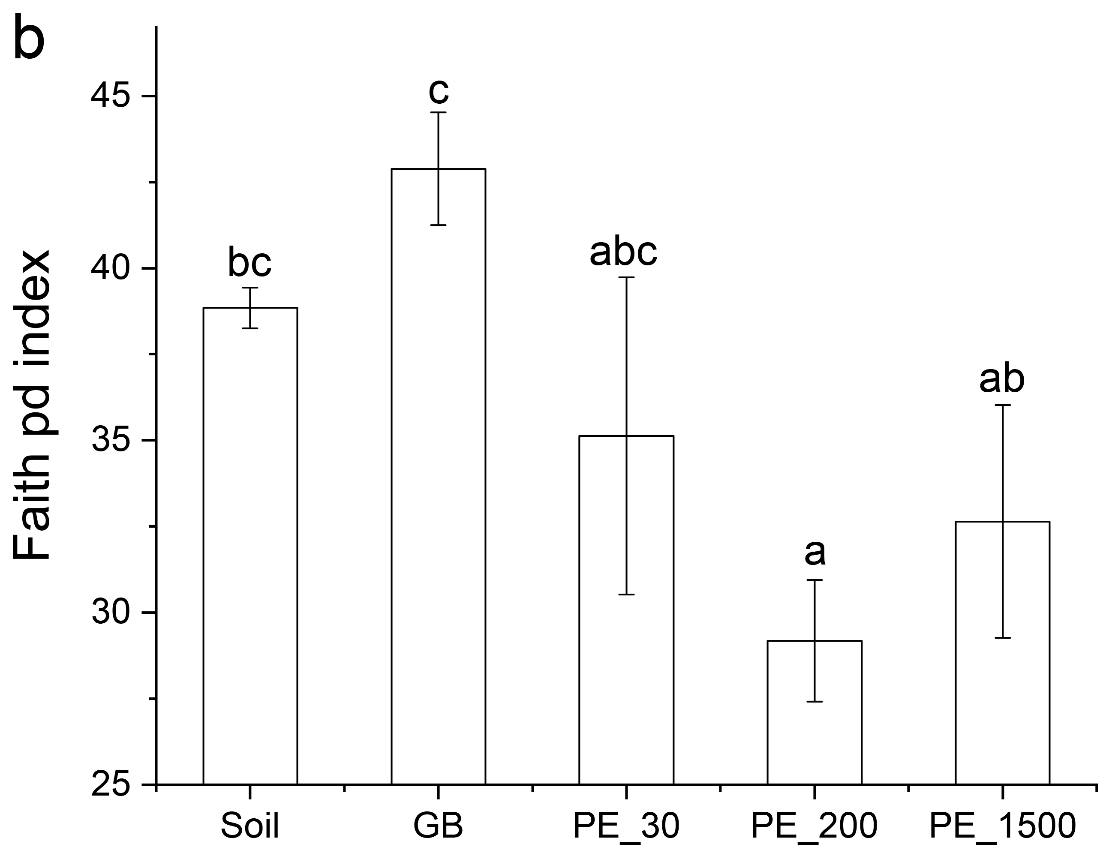


**Figure S4.** Comparison of alpha diversity (Shannon index: a; Faith pd index: b) of bacterial communities between different substrates. Significance of results was evaluated using ANOVA with Duncan test and labeled using different letters (significant level *p* < 0.05).


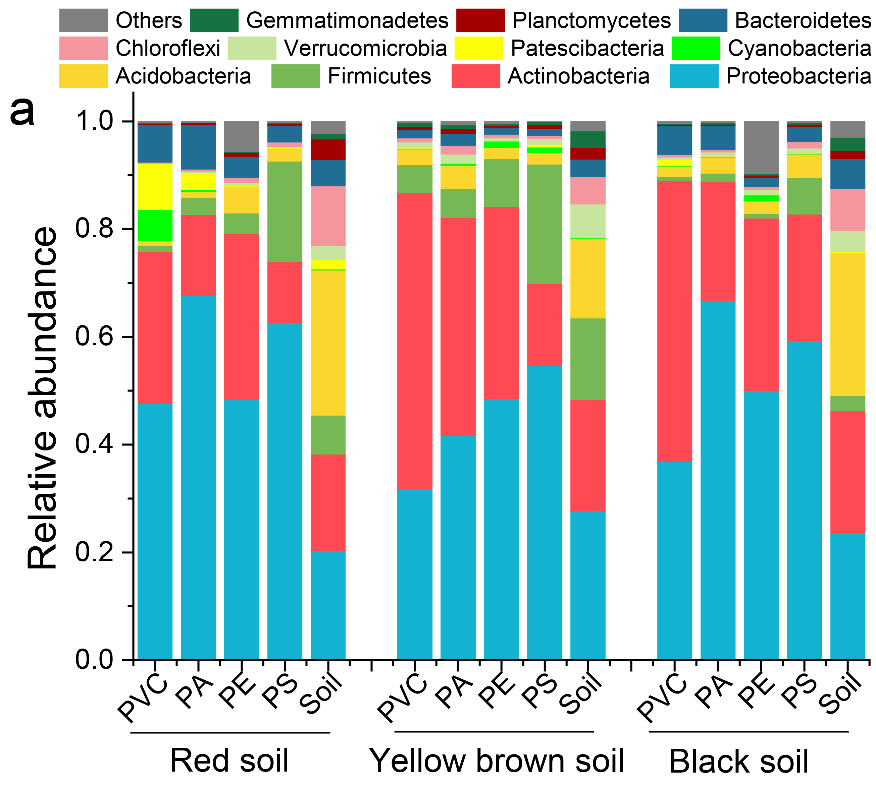


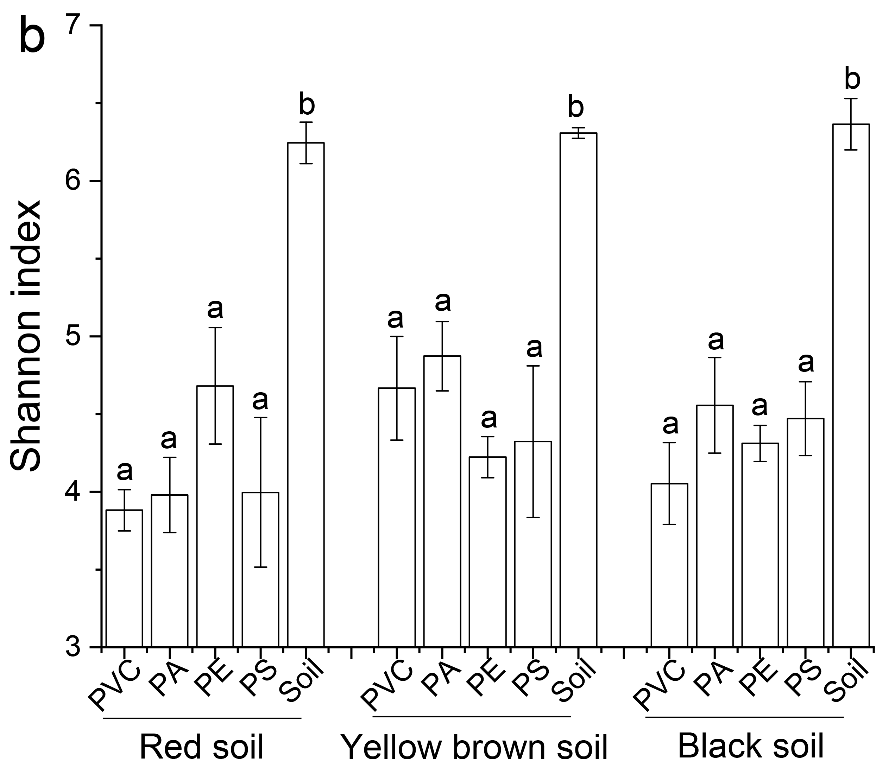


**Figure S5.** a) The composition of bacterial communities in different plastisphere and soil samples at the Phylum level. b) The alpha diversity (Shannon index) of bacterial communities in each treatment (mean ± SE; n = 5). The different letter indicated significant difference between different samples from the same soil environment (significant level *p* < 0.05).


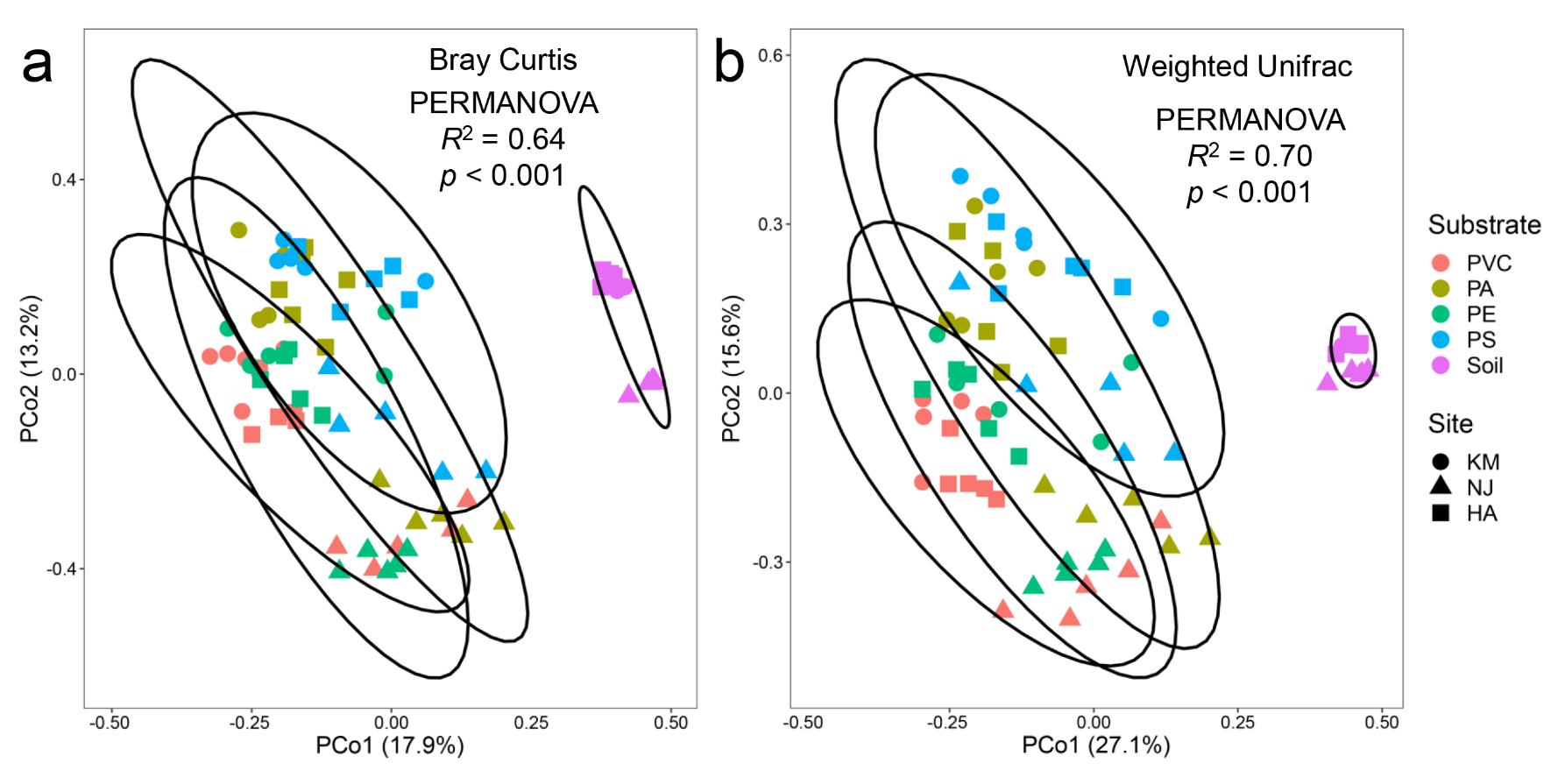


**Figure S6.** Principal coordinates analysis (PCoA) with a 95% confidence ellipse presenting the distribution of sample bacterial communities based on the Bray Curtis (a) and Weighted Unifrac (b) distances. Different shapes and colors represented different types of samples. The variation explained by the PCoA axes was listed in parentheses. The PERMANOVA was used to test significant difference (significant level *p* < 0.05).


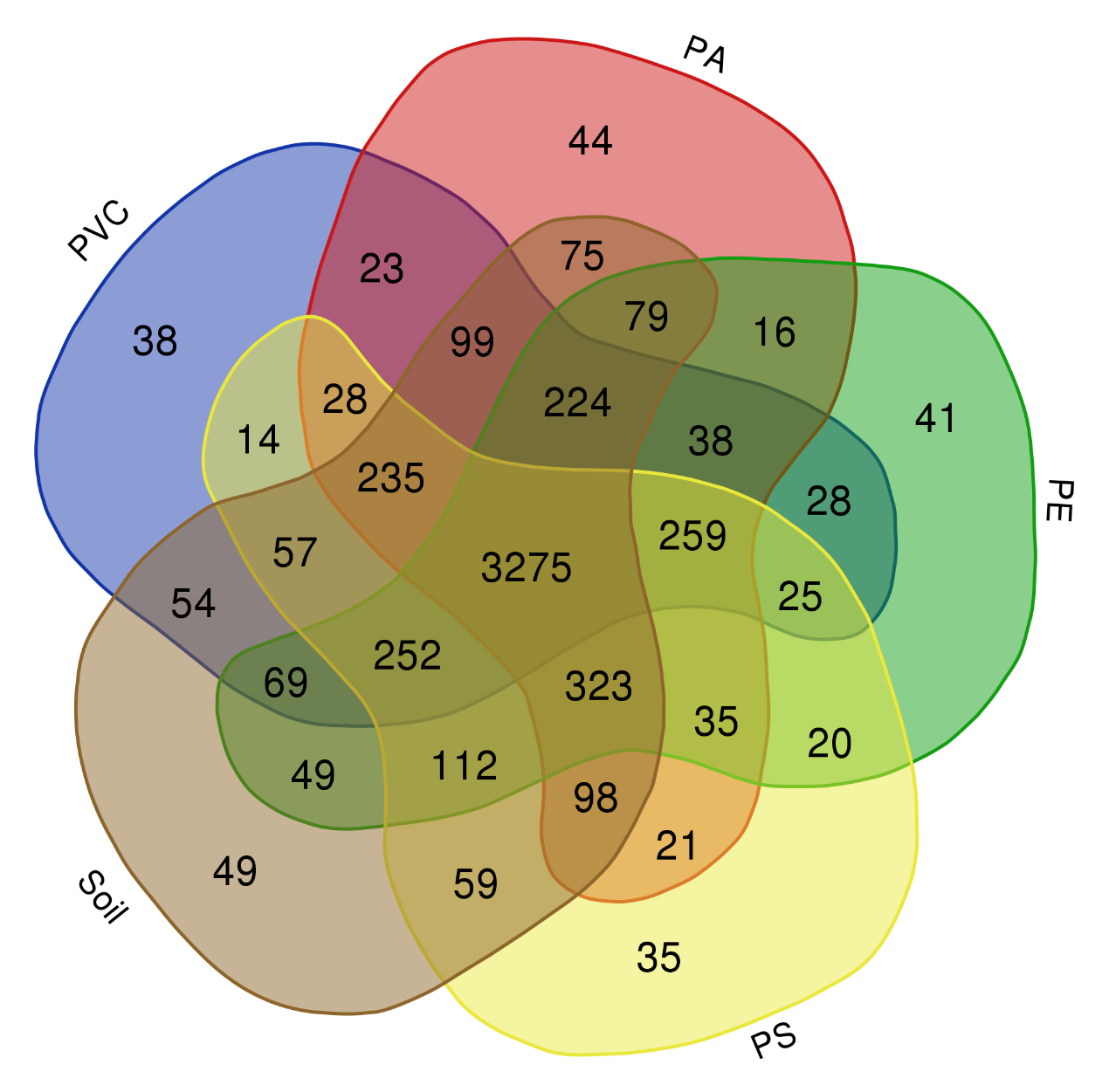


**Figure S7.** Venn diagram revealing the number of shared bacterial zOTUs among different plastispheres and soil.


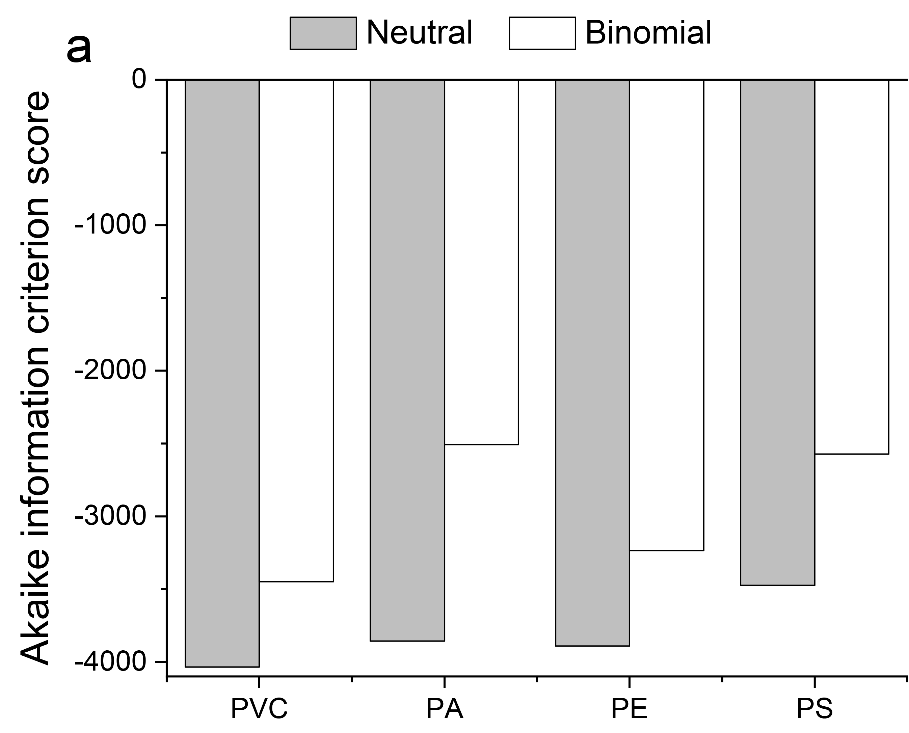


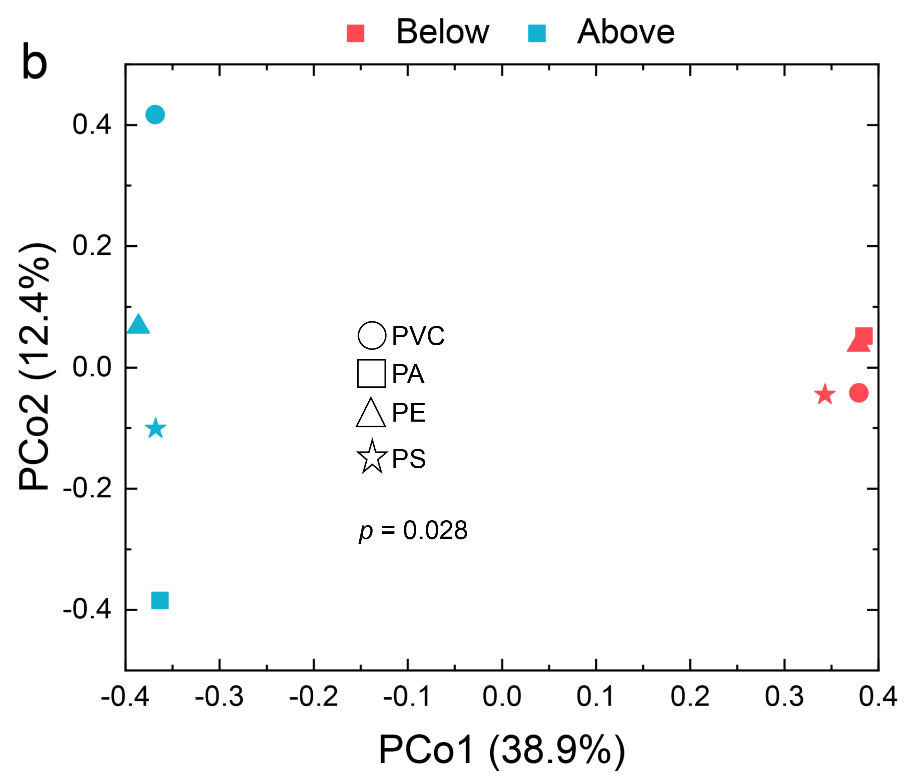


**Figure S8.** Characteristics of neutral models of bacterial communities for each plastisphere. (a) The comparison of Akaike information criterion (AIC) scores between a neutral model fit and fit of a binomial model. (b) Principal coordinates analysis presenting the distribution of bacterial communities of partitions above and below neutral model predictions.

**
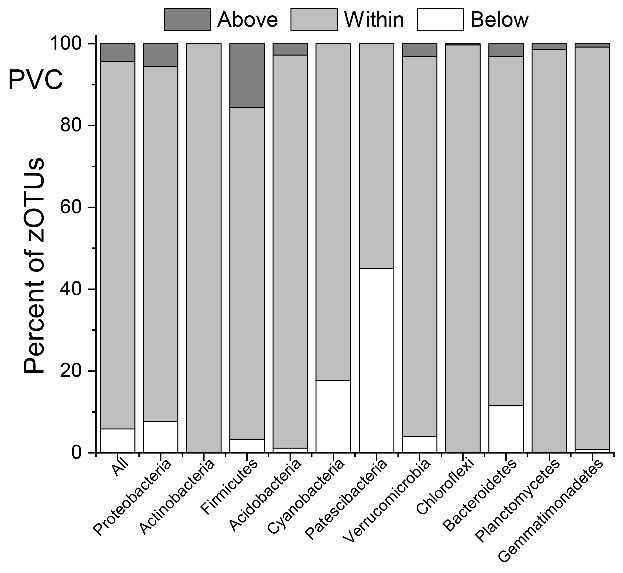

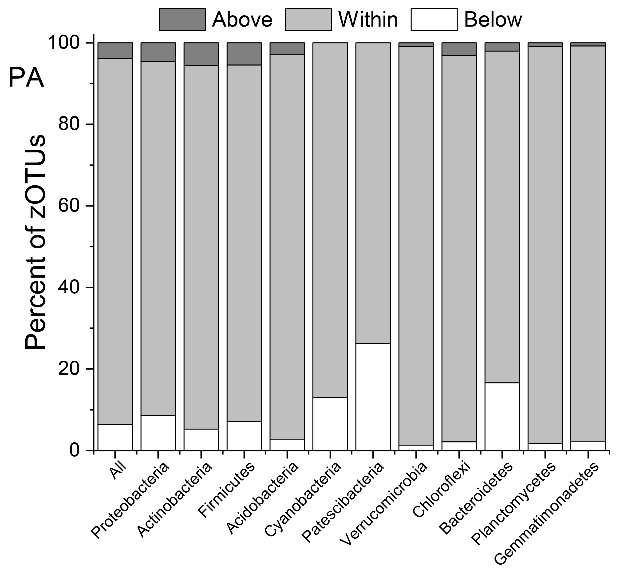
**

**
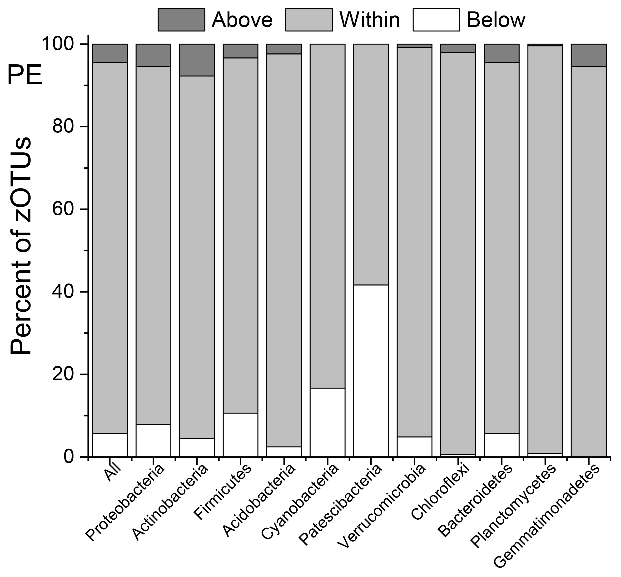

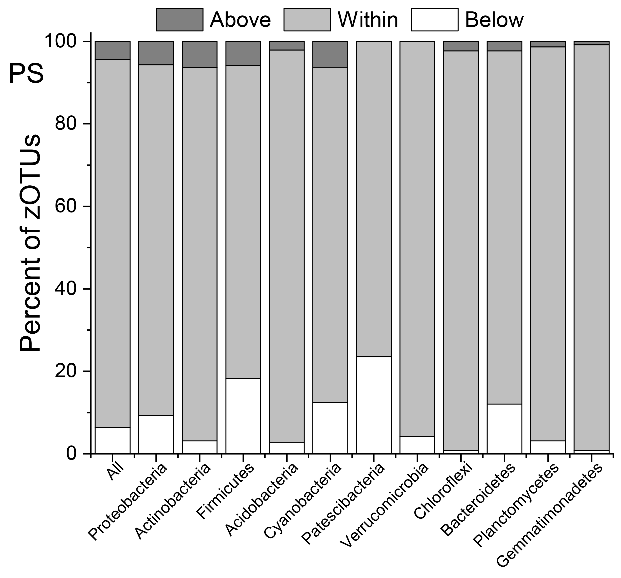
**

**Figure S9.** The percent of zOTUs from each plastisphere (Phylum level) that fall within, below and above neutral model prediction.

**
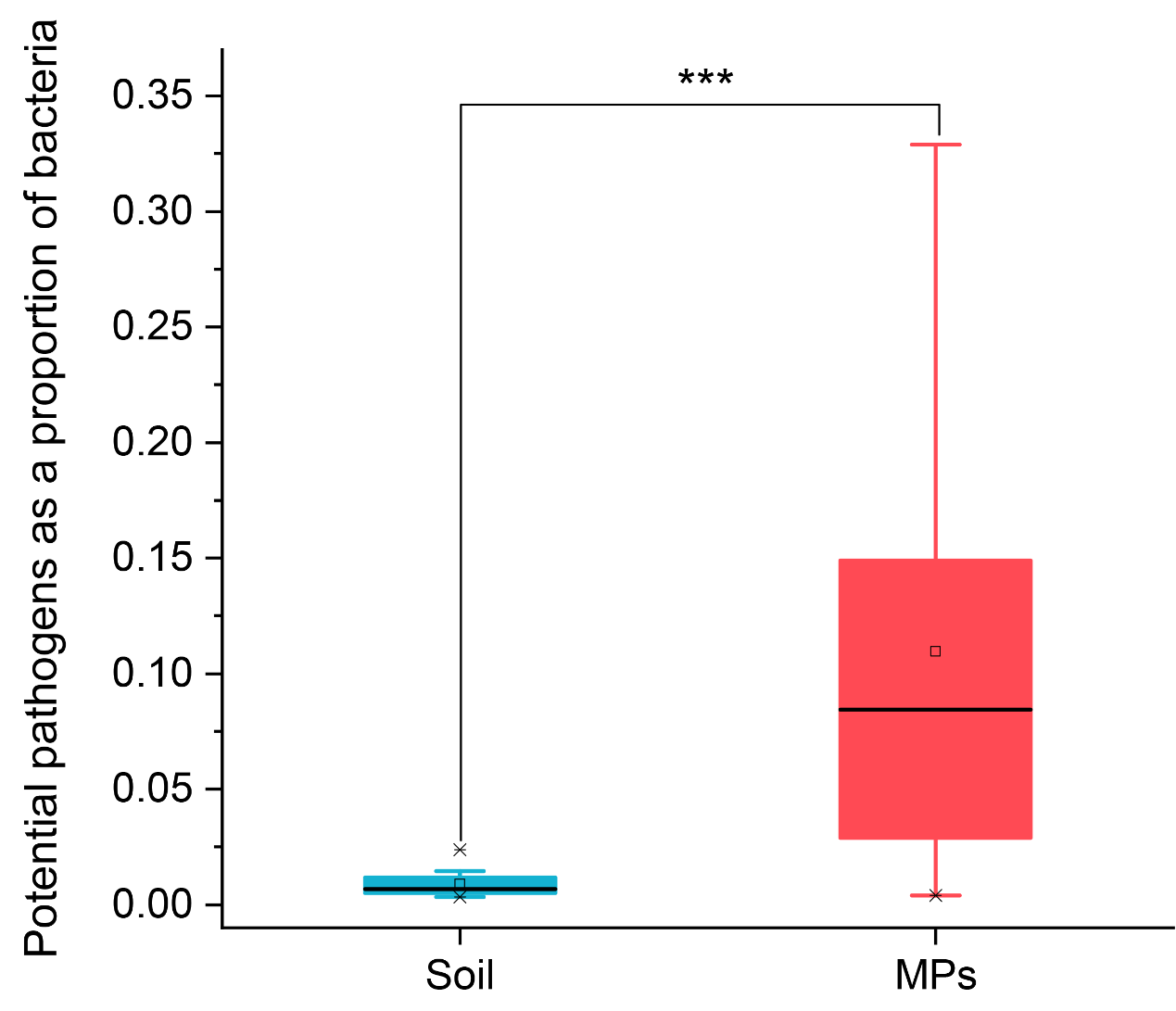
**

**Figure S10.** Ratio of potential bacterial pathogens/bacteria detected in the plastisphere and soil samples. The “***” indicated *p* < 0.001.

**
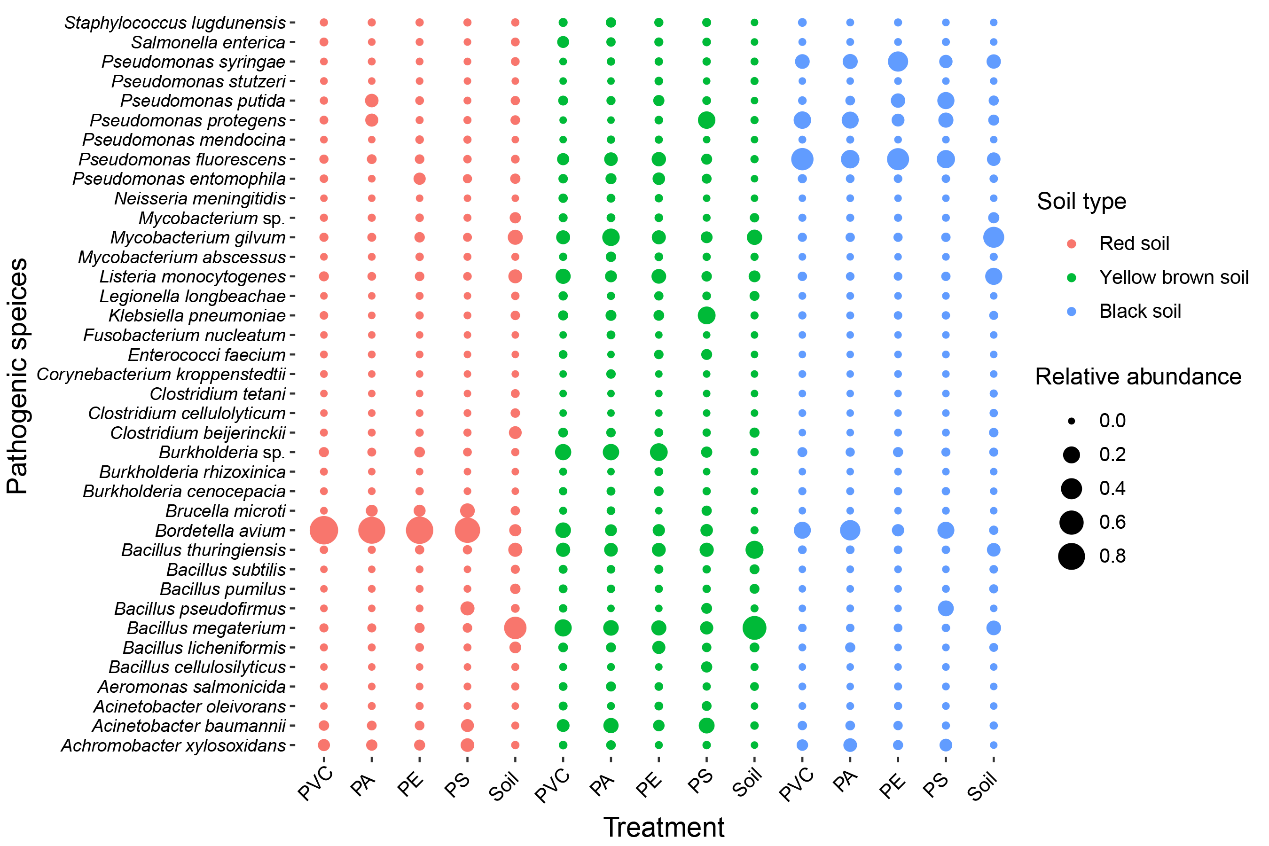
**

**Figure S11.** Bubble diagram presenting relative abundance of potential bacterial pathogenic species.

**
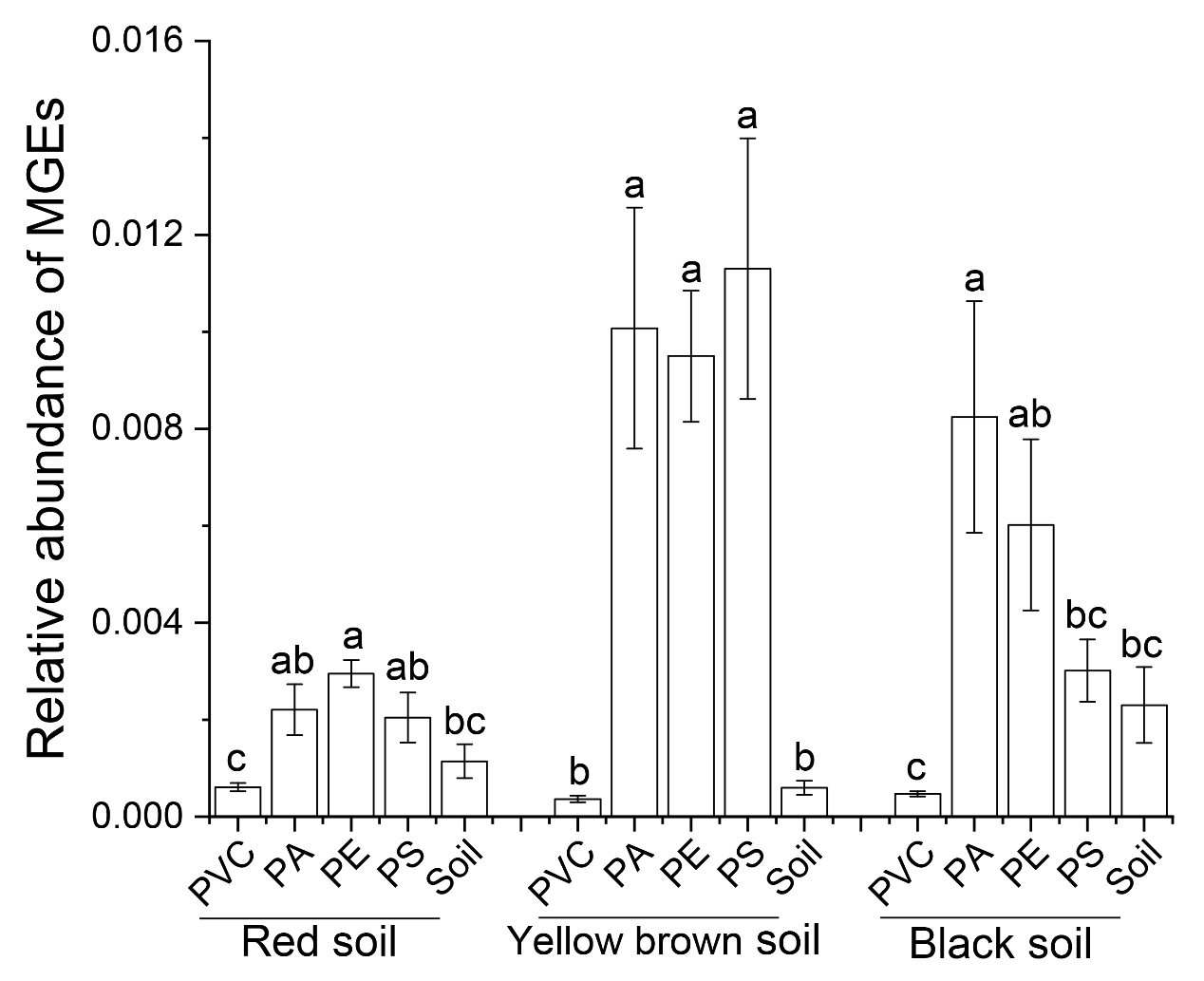
**

**Figure S12.** Relative abundance of MGEs in different plastisphere and soil samples (Mean ± SE; n = 5). The different letter indicated significant difference between different samples from the same soil environment.

**
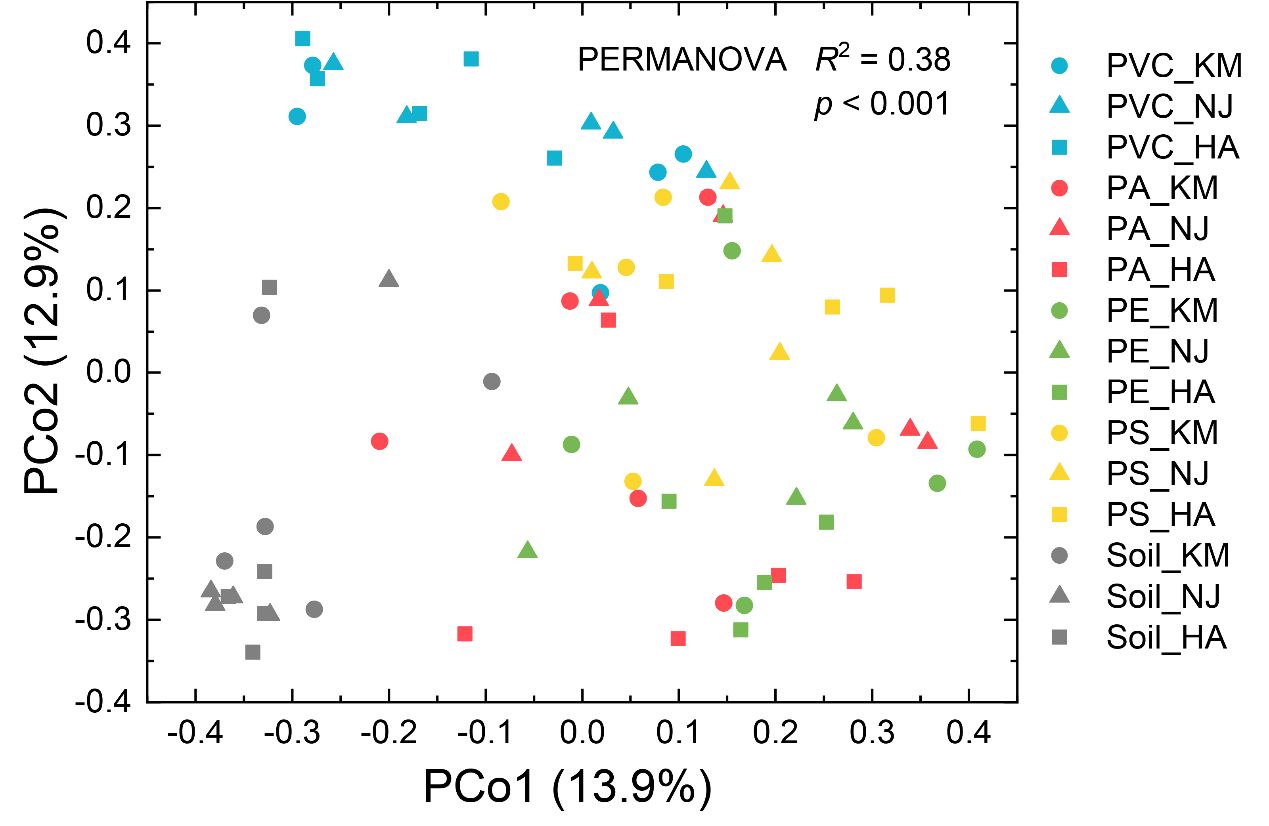
**

**Figure S13.** Principal coordinates analysis (PCoA) presenting the ARG profiles using the Bray-curtis distance. Different shapes and colors represented different types of samples. The variation explained by the PCoA axes was listed in parentheses. Significant analysis of variance used distance matrices (PERMANOVA) via the Adonis test (999 permutations).

**
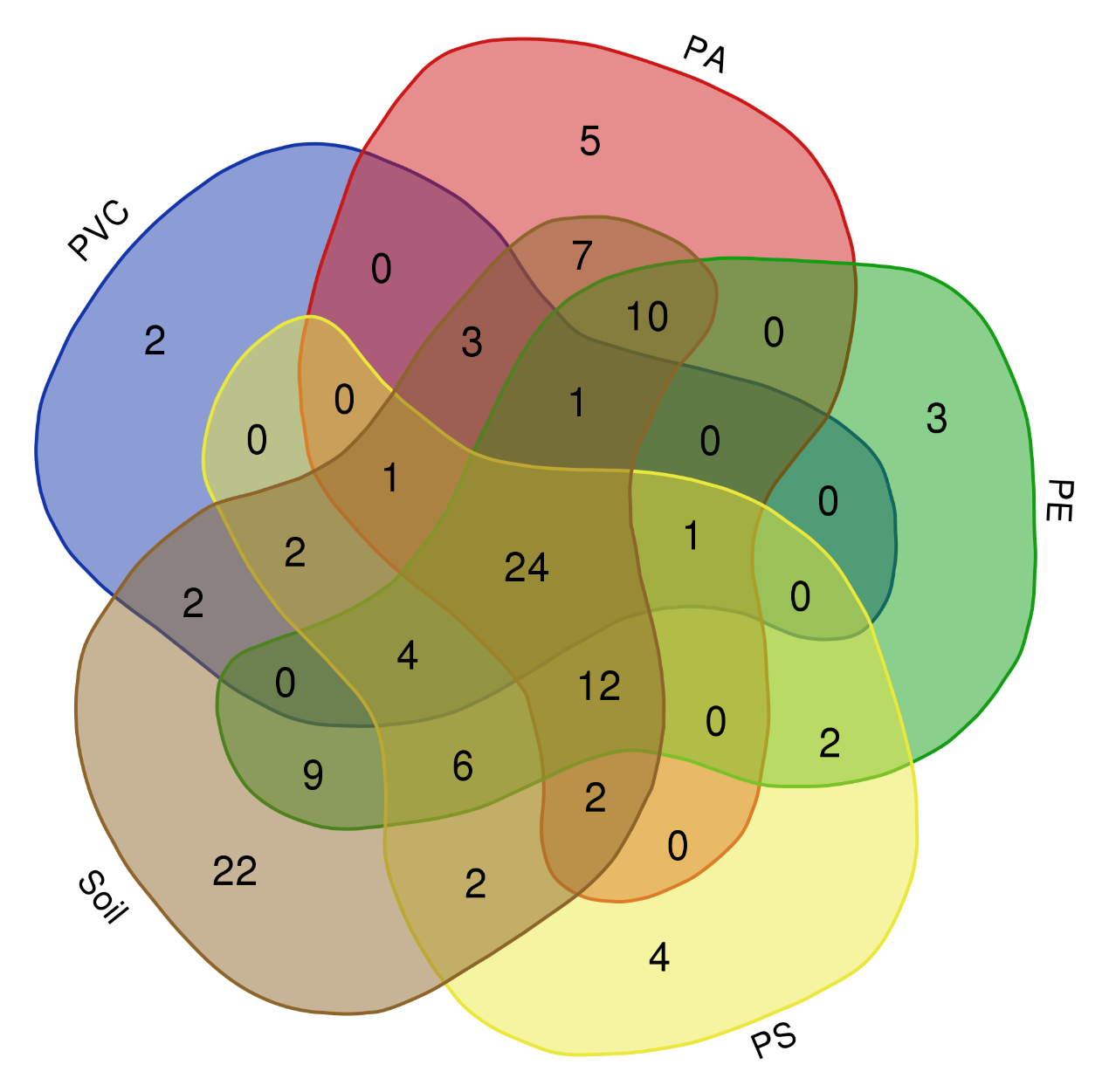
**

**Figure S14.** Venn diagram revealing the number of shared ARGs among different plastispheres and soil.

**
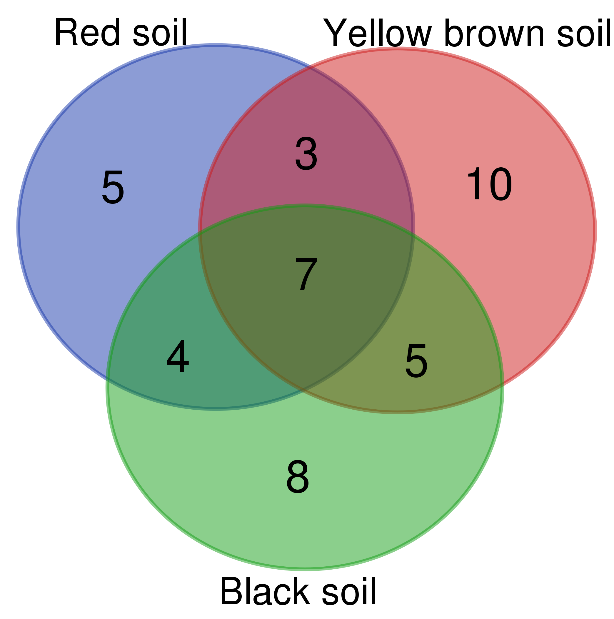

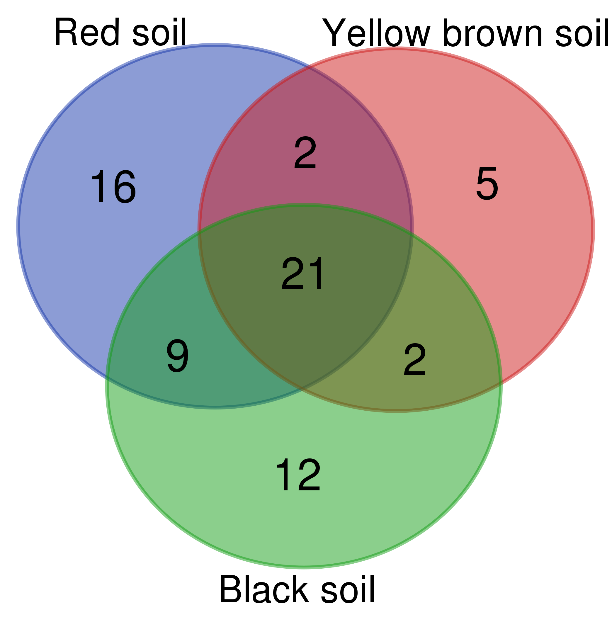
**

**PVC**

**PA**

**
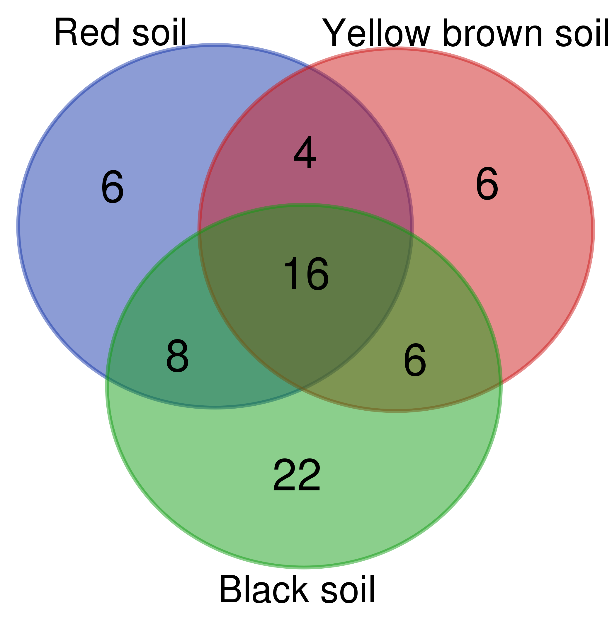

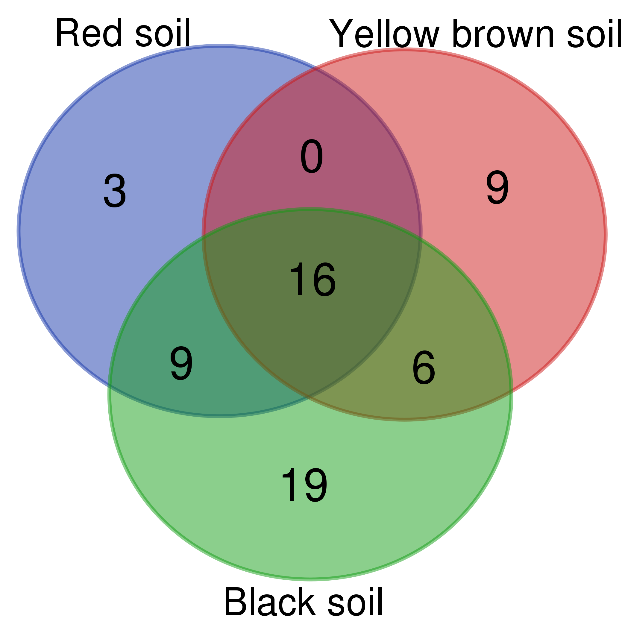
**

**PS**

**PE**

**Figure S15.** Venn diagram revealing the number of shared ARGs among the same plastisphere from different soil environments.

**
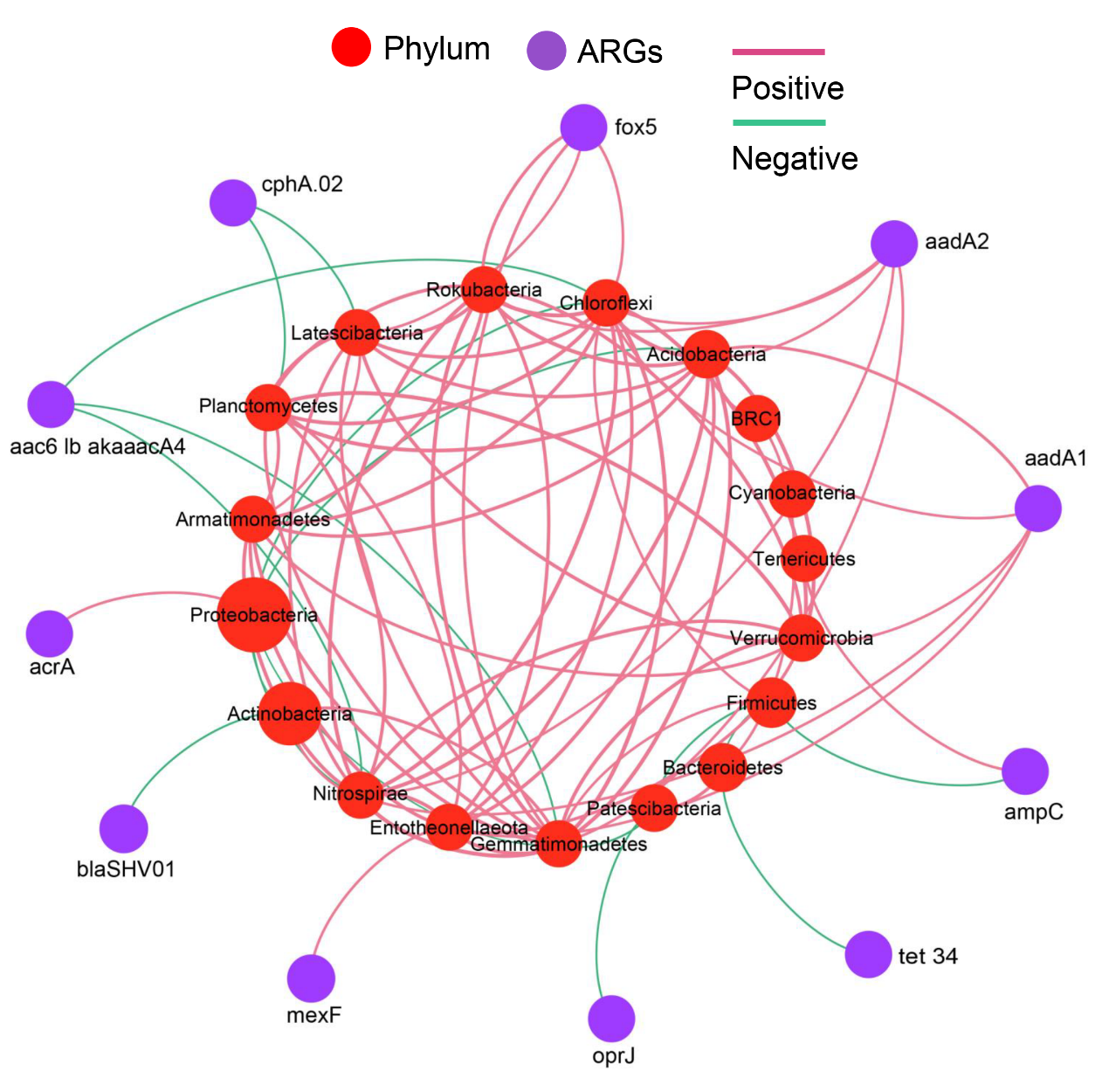
**

**Figure S16.** Co-occurrence network of ARGs and bacterial communities (Phylum level) in the plastisphere.

**
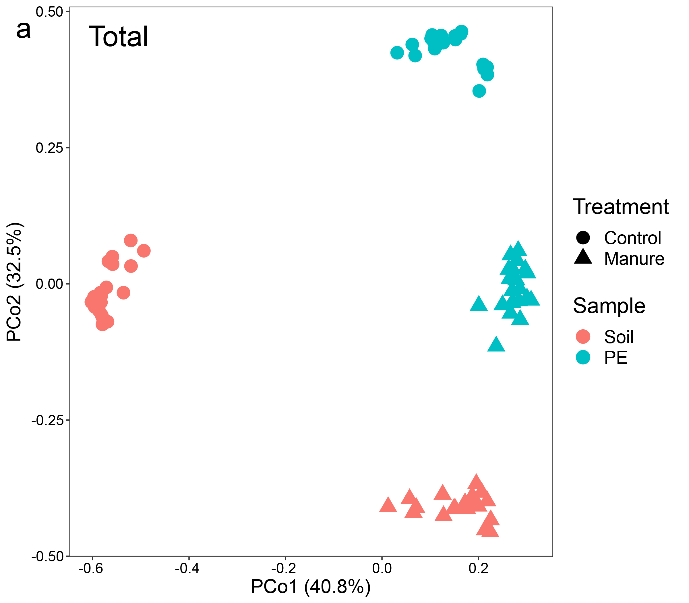

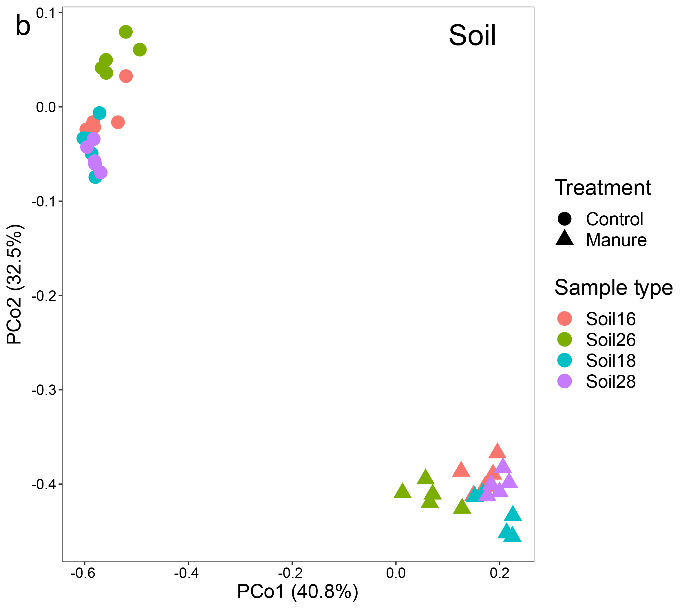

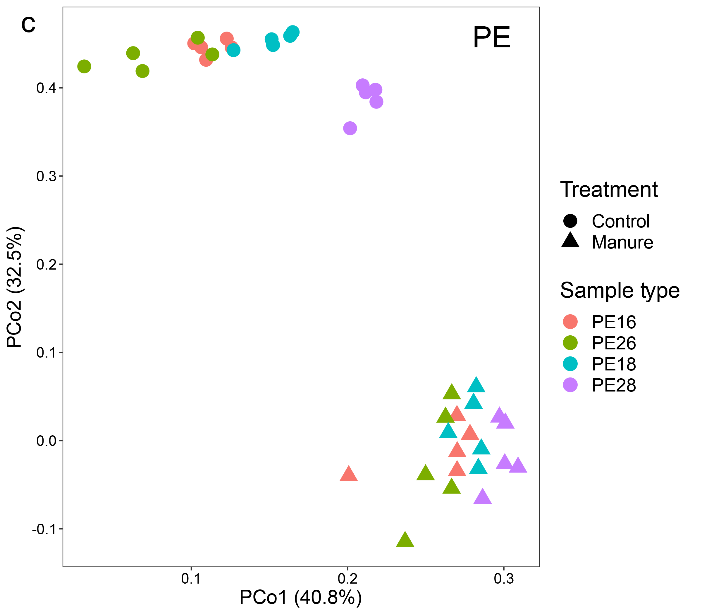
**

**Figure S17.** Principal coordinates analysis (PCoA) presented the distribution of ARG profiles from different treatments based on the Bray Curtis distance.

**
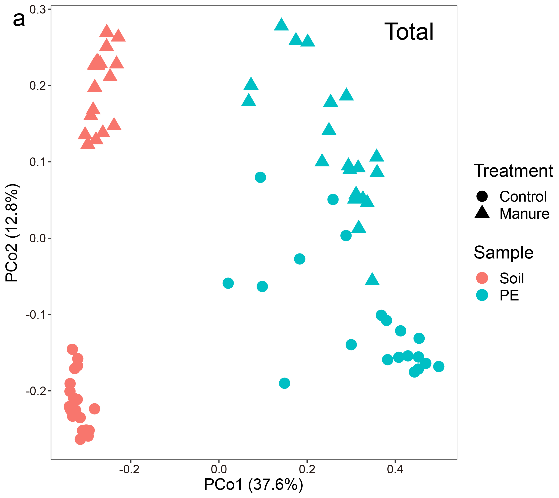

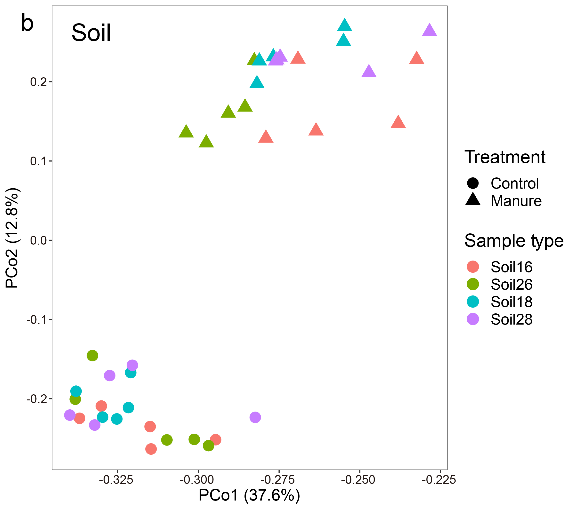

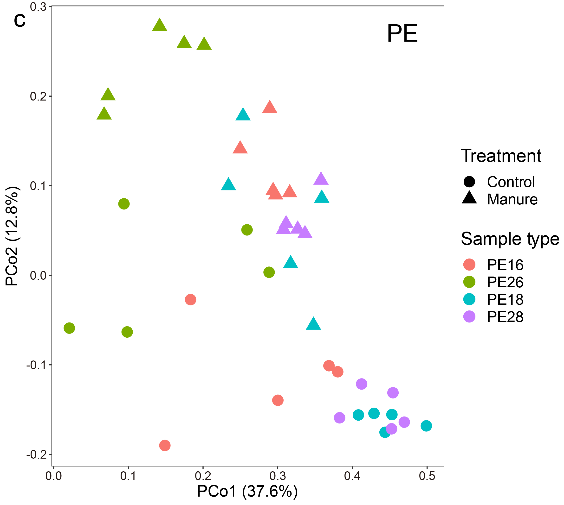
**

**
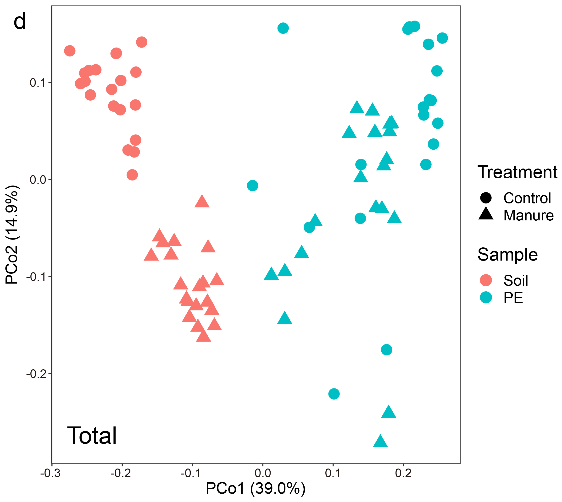

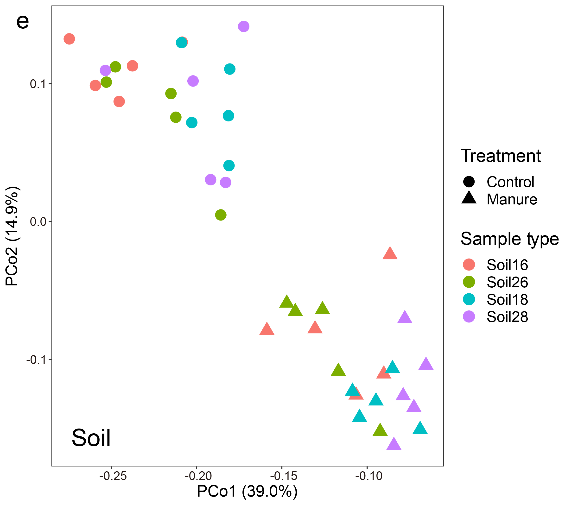

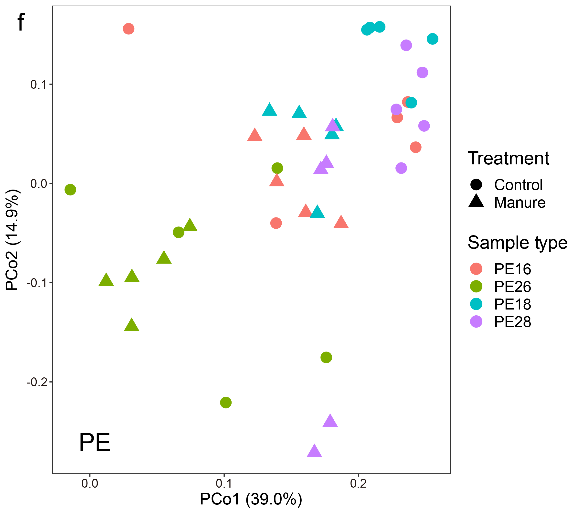
**

**Figure S18.** Principal coordinates analysis (PCoA) presented the distribution of bacterial communities from different treatments based on the Bray Curtis (a, b and c) and Weighted Unifrac (d, e and f) distances.

**
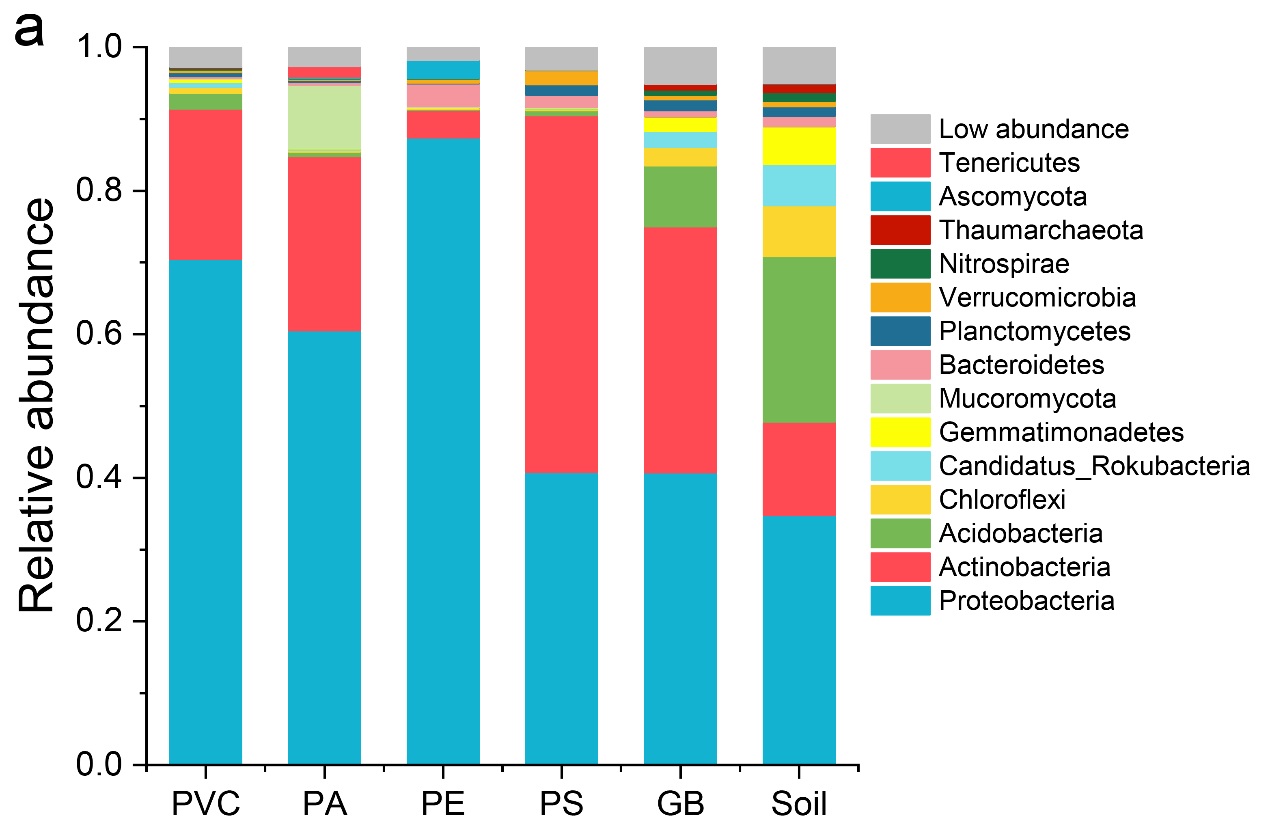
**

**
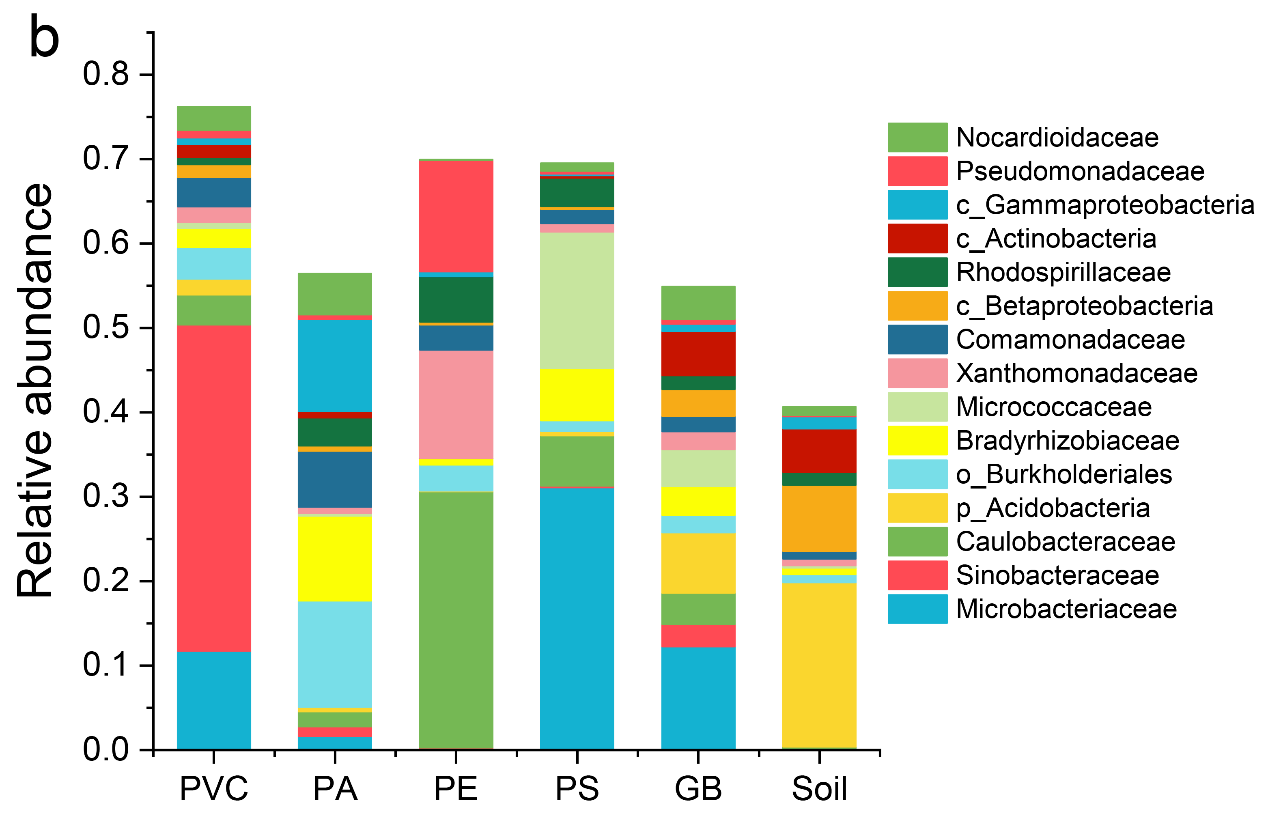
**

**Figure S19.** The composition of microbial communities (Phylum level: a; Family level: b) in the plastisphere from different substrates.

**
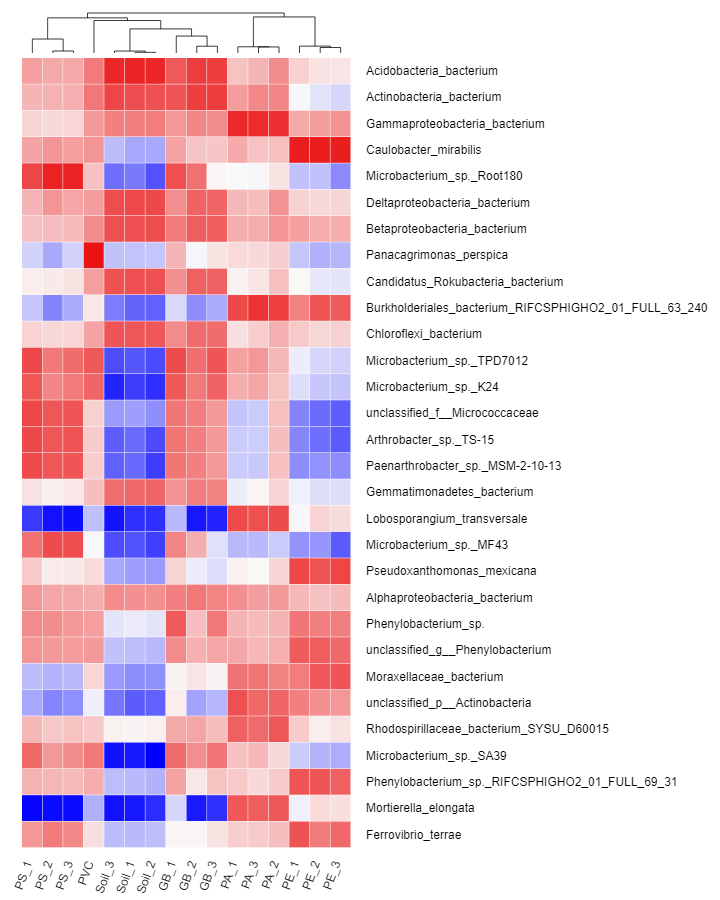
**

**Figure S20.** Heat map revealing the 30 most abundant species in the plastisphere.

**
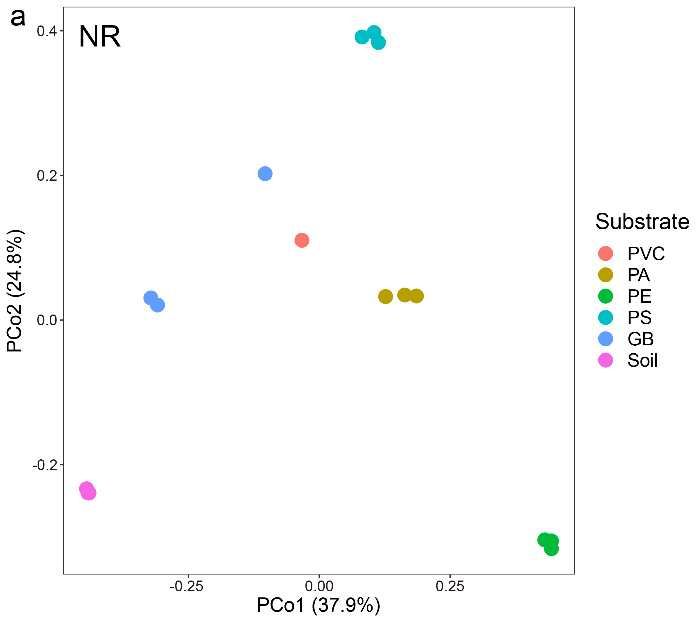

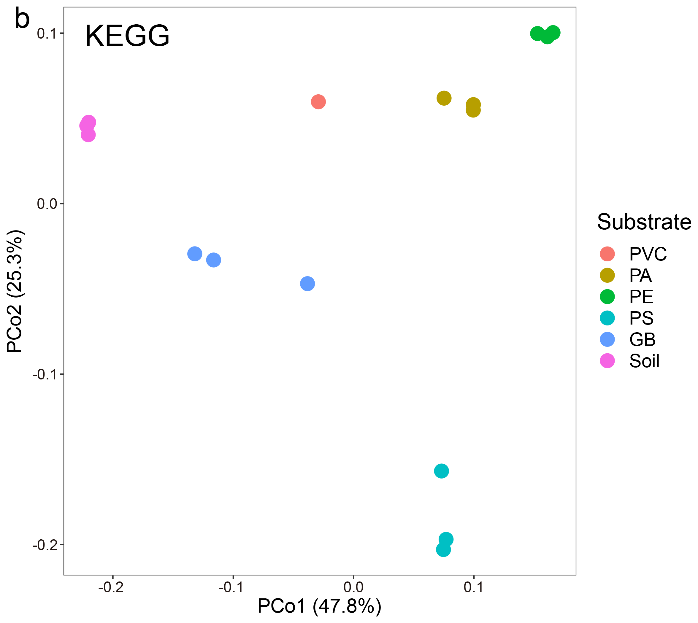

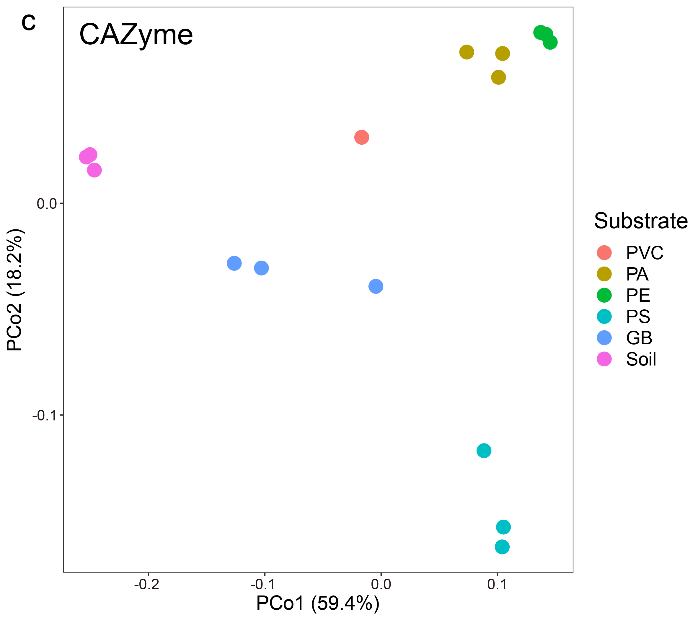
**

**Figure S21.** Principal coordinates analysis (PCoA) revealing the distribution of microbial communities (a) and functions (b and c) from different substrates based on the Bray Curtis distance.

**
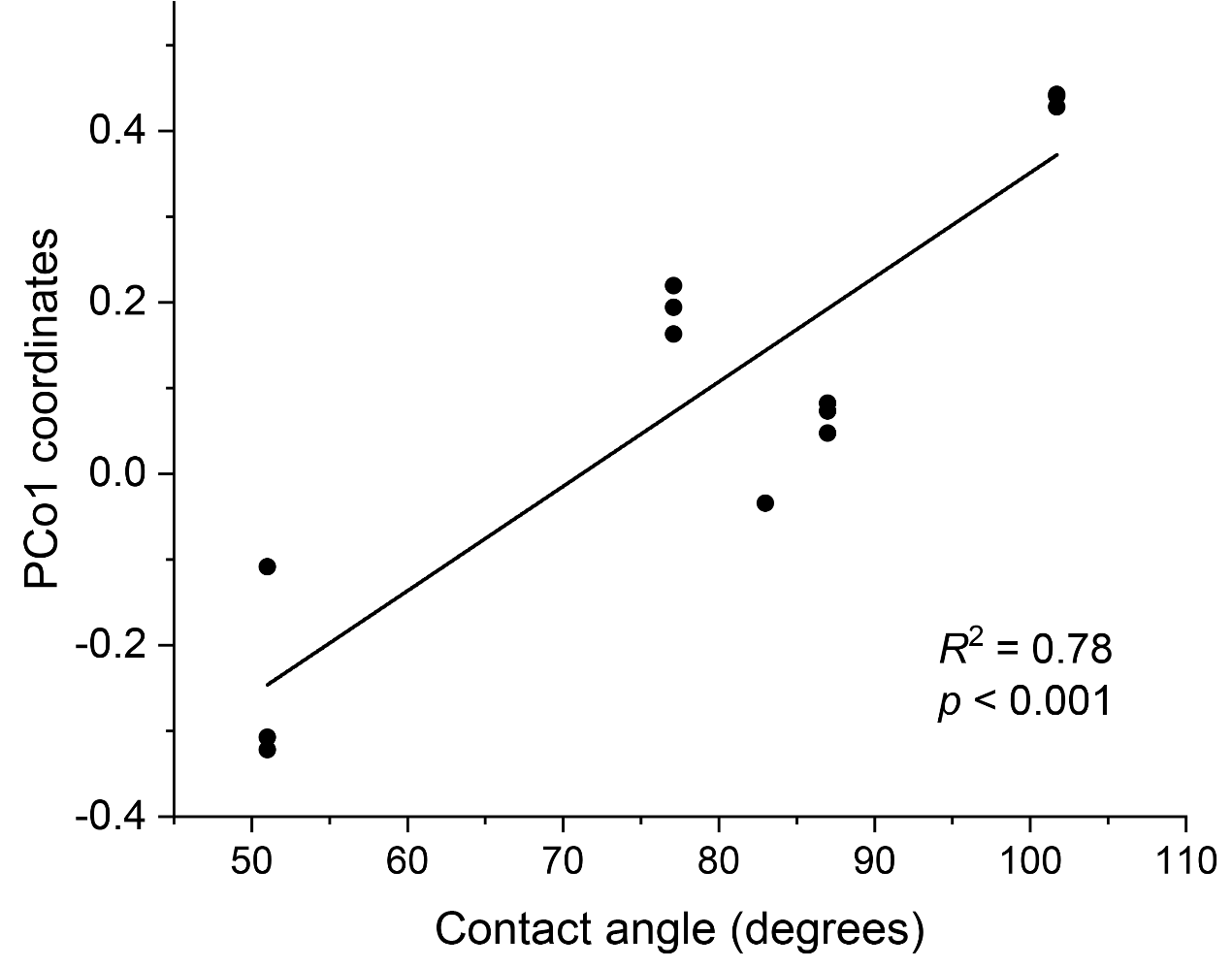
**

**Figure S22.** Community structure defined as multivariate ordination along the first principal component (PCo1) axis for Bray–Curtis distance as a function of substrate hydrophobicity (contact angle).


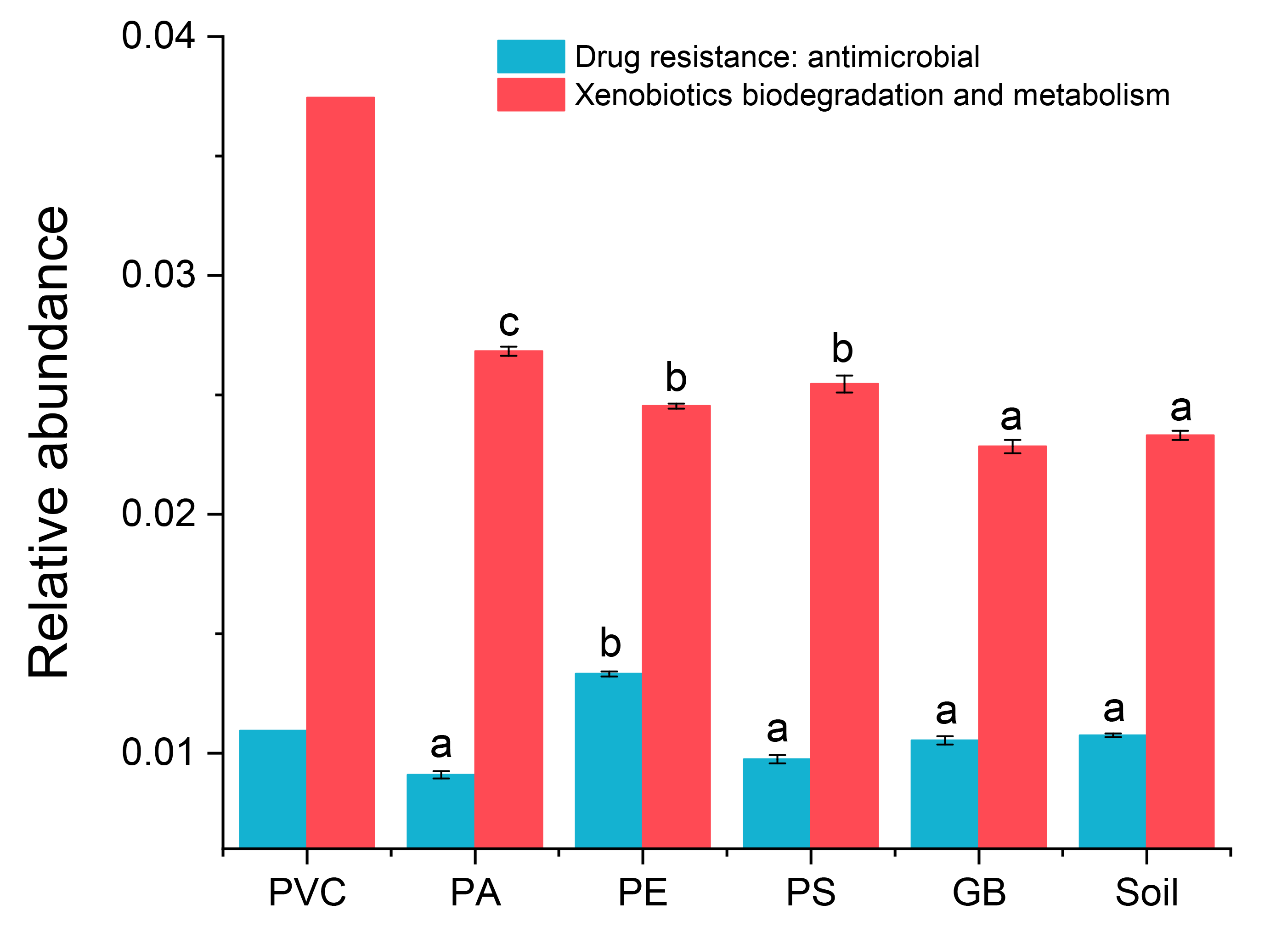


**Figure S23.** Two KEGG pathways from different substrates (PVC, PA, PE, PS, GB and Soil).

**
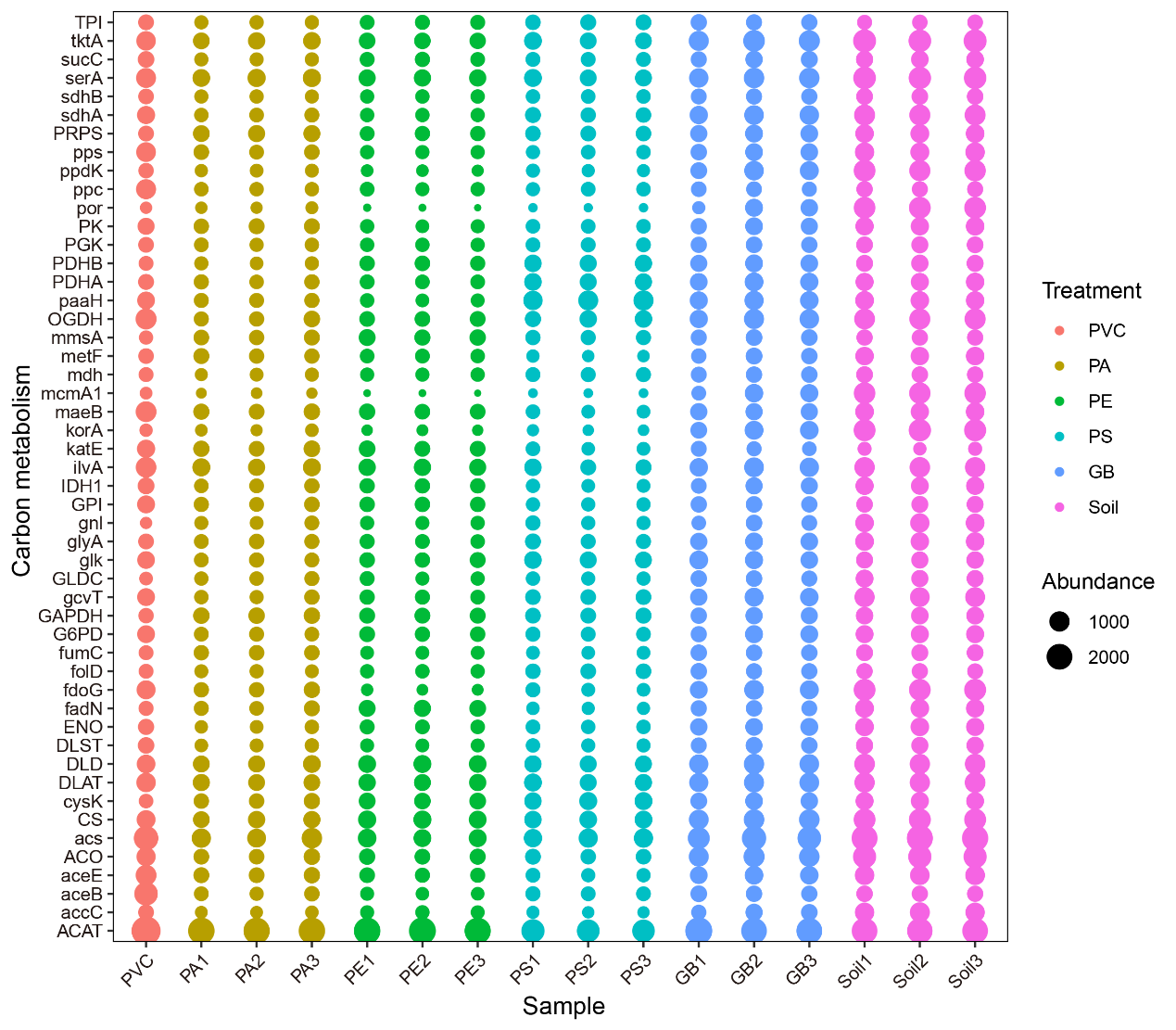
**

**Figure S24.** Bubble Plot revealing profiles of the carbon metabolism related KOs based on KEGG from different substrates (PVC, PA, PE, PS, GB and Soil).

**
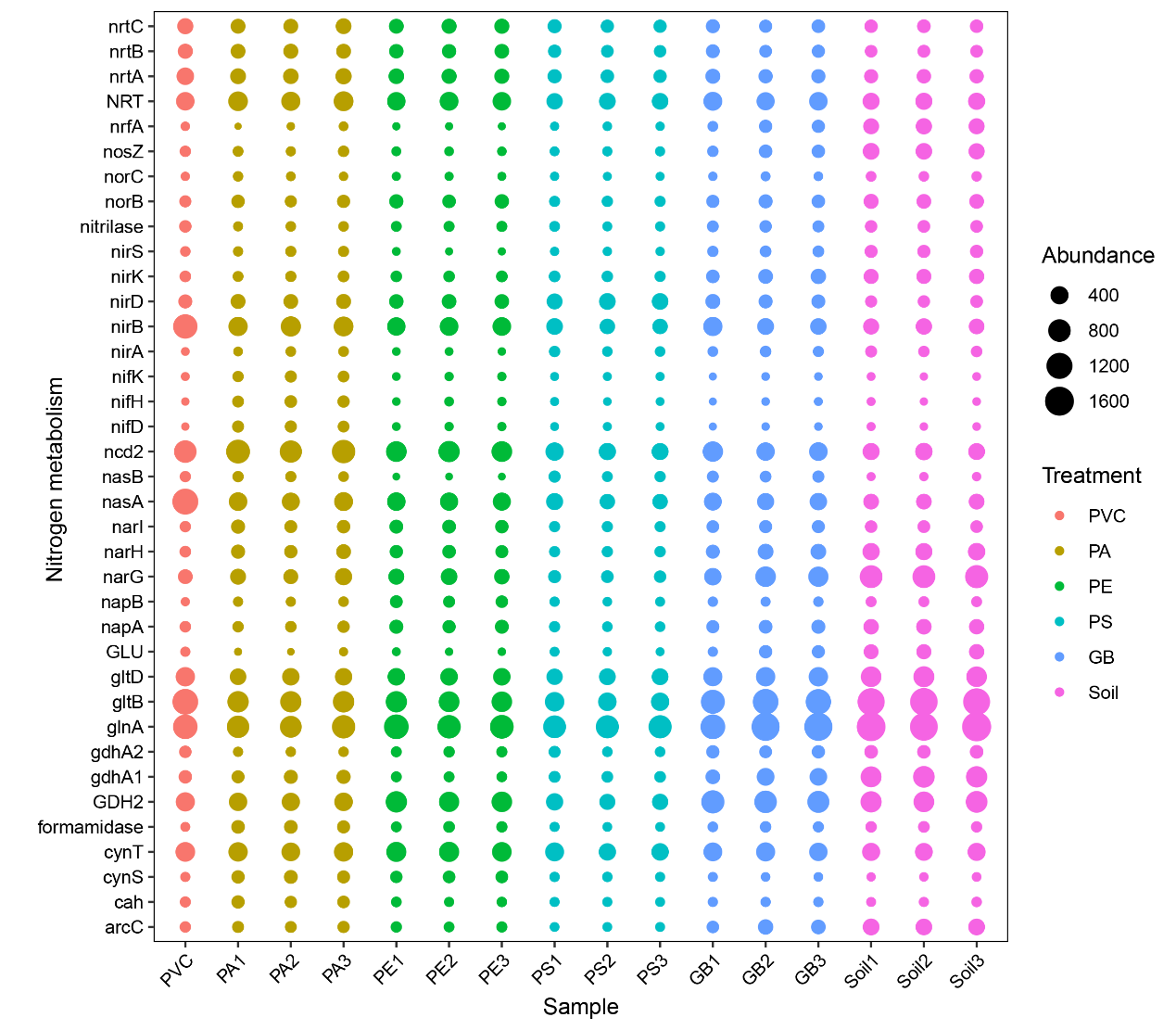
**

**Figure S25.** Bubble Plot revealing profiles of the Nitrogen metabolism related KOs based on KEGG from different substrates (PVC, PA, PE, PS, GB and Soil).

**
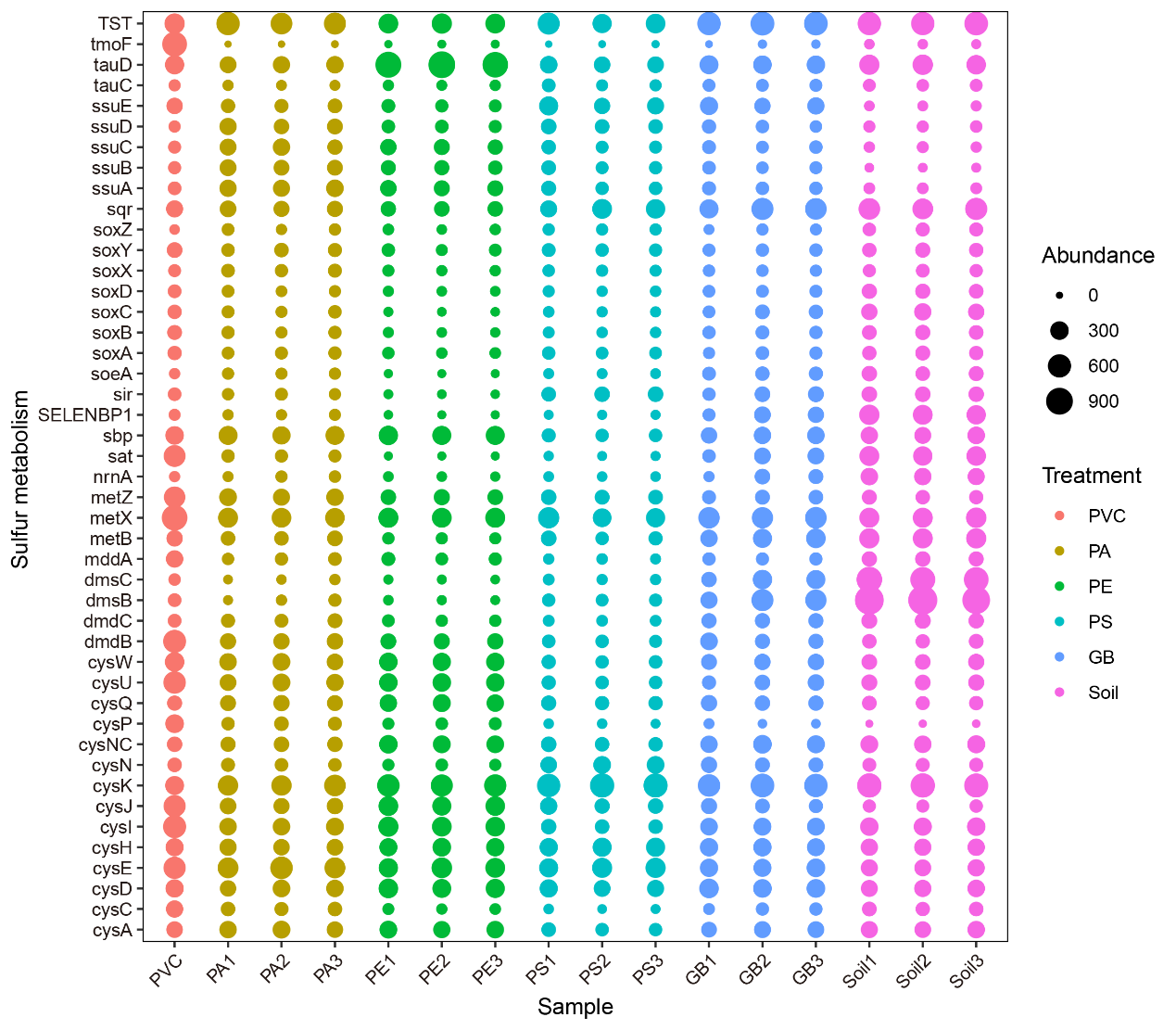
**

**Figure S26.** Bubble Plot revealing profiles of the Sulfur metabolism related KOs based on KEGG from different substrates (PVC, PA, PE, PS, GB and Soil).

**
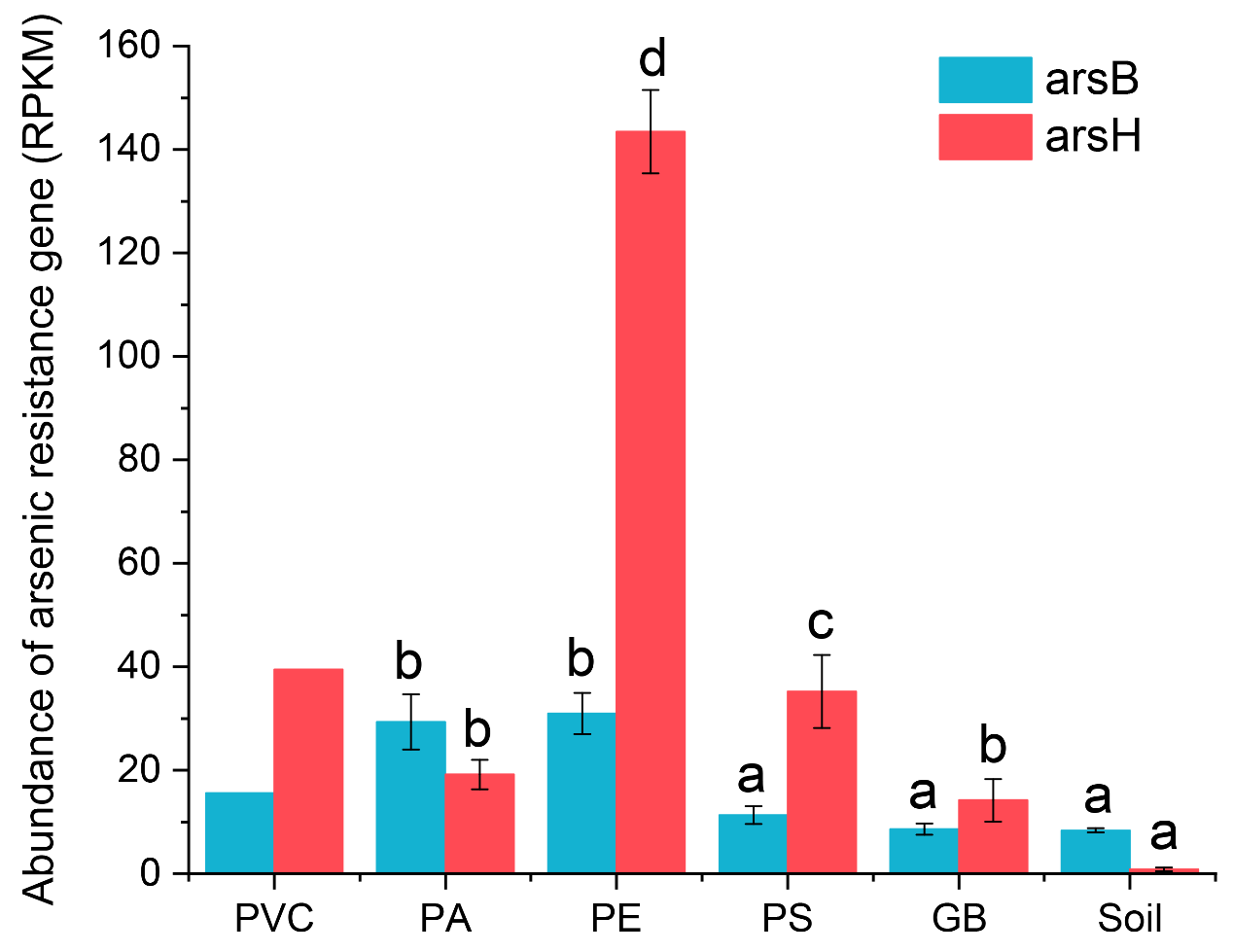
**

**Figure S27.** Abundance of arsenic resistance gene related KOs based on KEGG in different substrates (PVC, PA, PE, PS, GB and Soil).

**
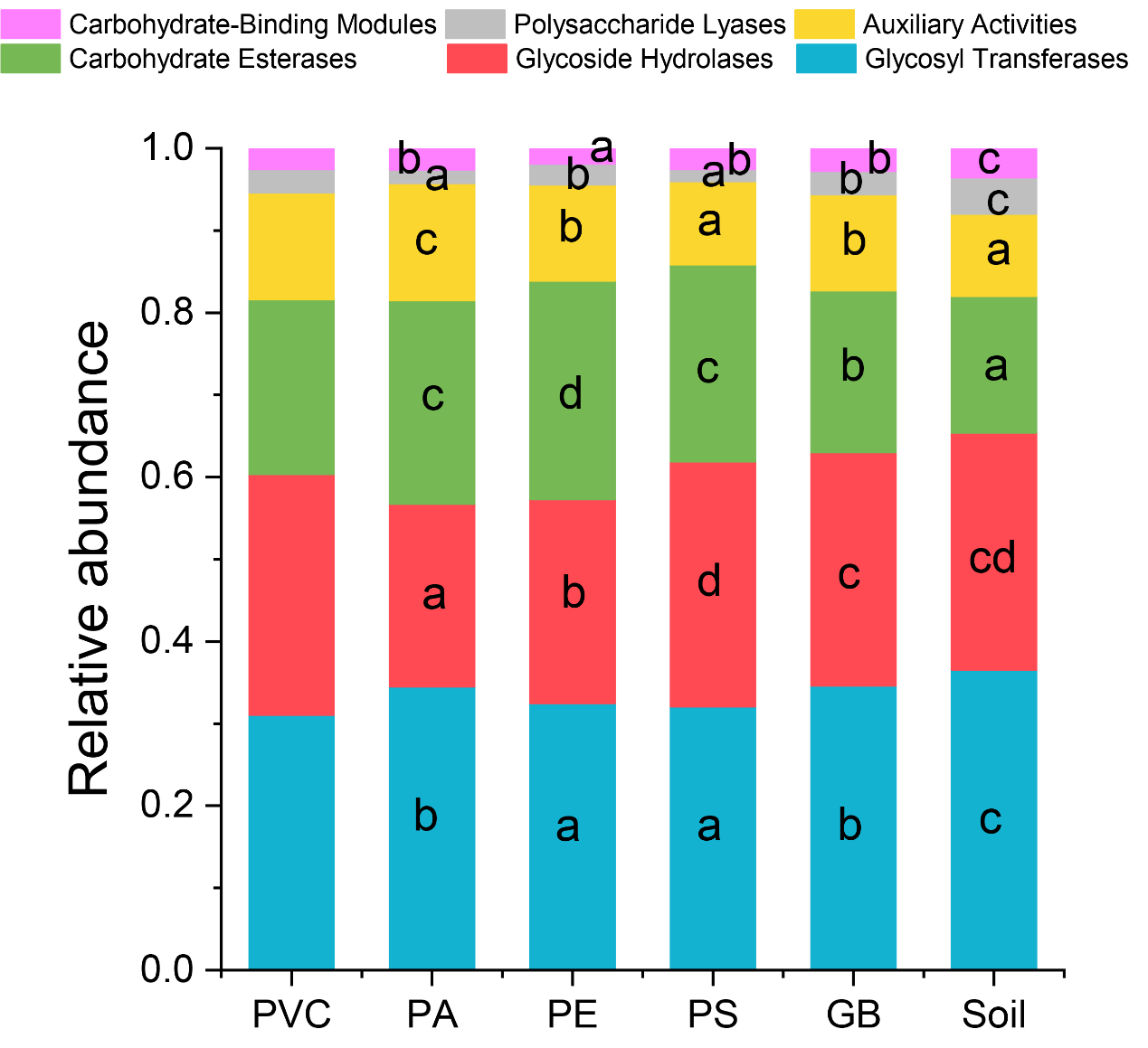
**

**Figure S28.** Functional composition of microbial community based on CAZyme from different substrates (PVC, PA, PE, PS, GB and Soil).

**
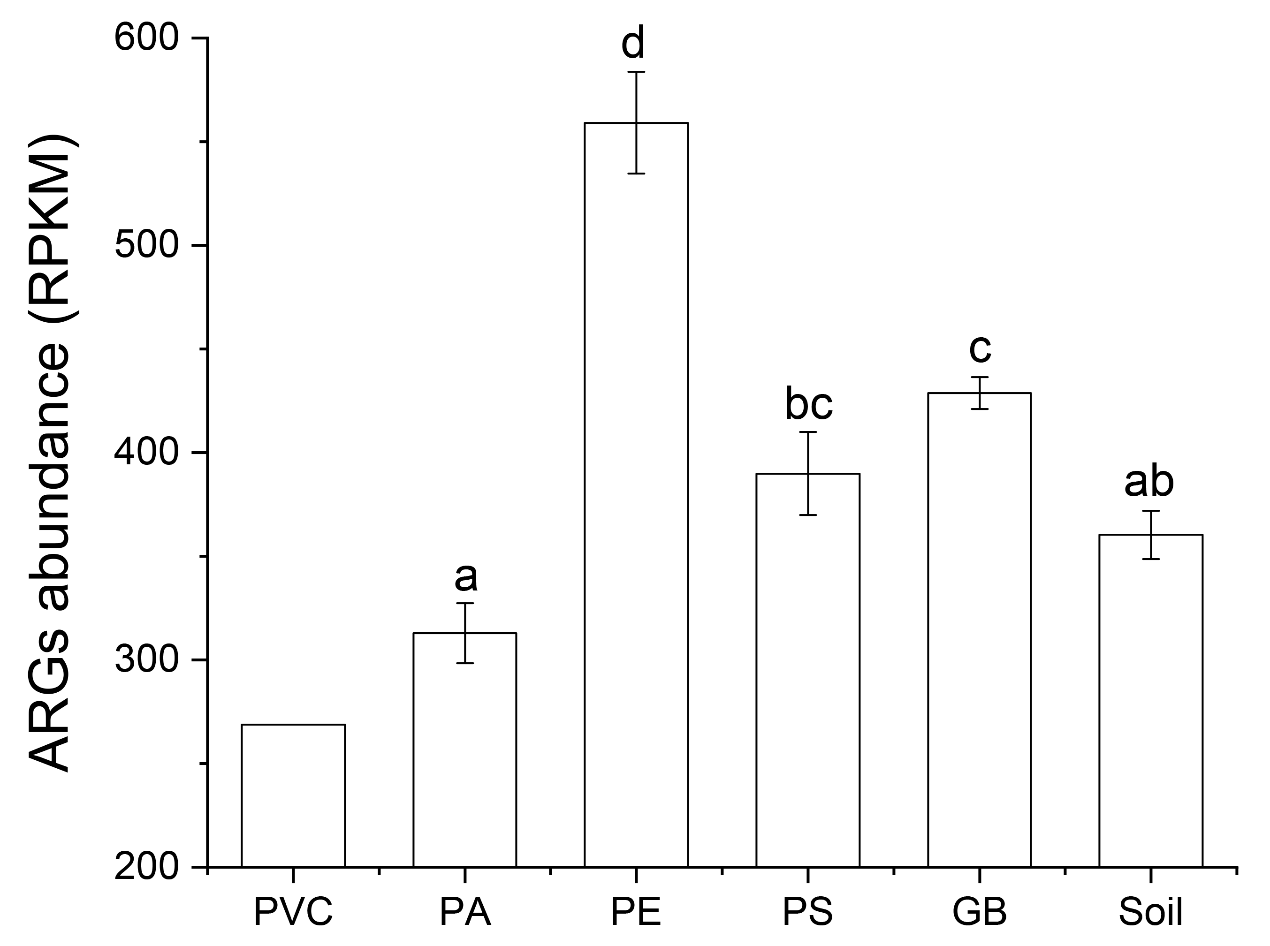
**

**Figure S29.** Abundance of ARGs (Mean; n = 3) in different substrates.

**
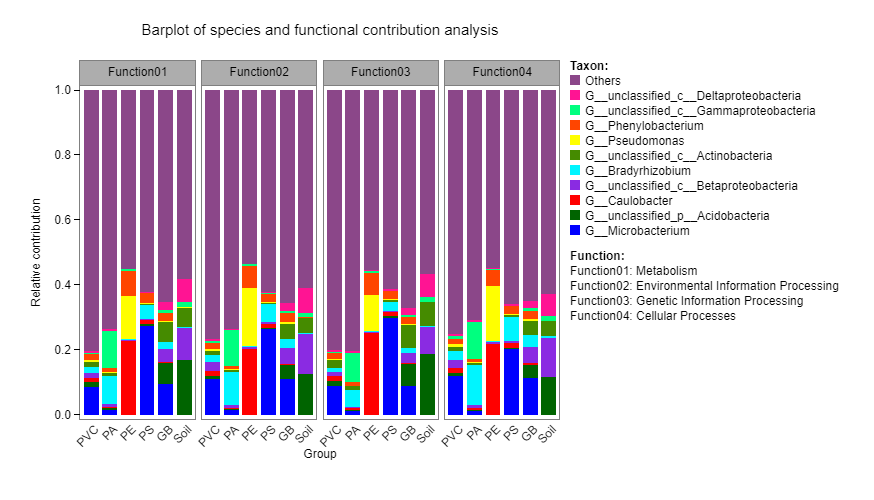

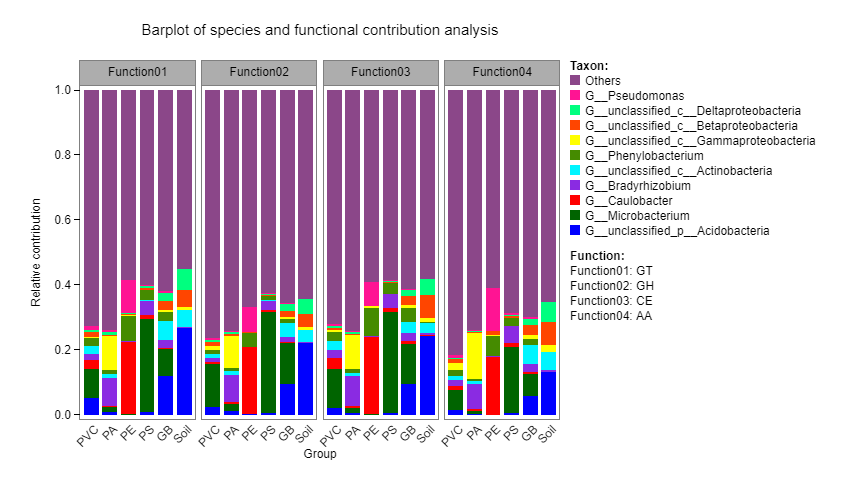
**

**a**

**b**

**Figure S30.** Contribution of the 10 most abundant genera to the 4 most abundant functions based on KEGG (a) and CAZyme (b) in each substrate.

**
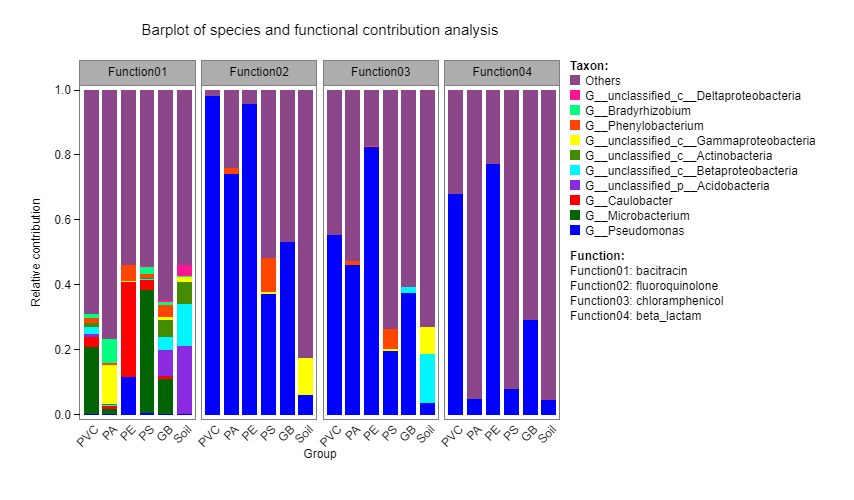
**

**Figure S31.** Contribution of the 10 most abundant genera to the 4 most abundant ARGs classes in each substrate.
